# Comprehensive Flow Cytometric Characterization of Bronchoalveolar Lavage Cells Indicates Comparable Phenotypes Between Asthmatic and Healthy Horses But Functional Lymphocyte Differences

**DOI:** 10.3389/fimmu.2022.896255

**Published:** 2022-07-06

**Authors:** A. Elisabeth Gressler, Sabrina Lübke, Bettina Wagner, Corinna Arnold, Katharina L. Lohmann, Christiane L. Schnabel

**Affiliations:** ^1^ Institute of Immunology, Faculty of Veterinary Medicine, Leipzig University, Leipzig, Germany; ^2^ Department of Population Medicine and Diagnostic Sciences, College of Veterinary Medicine, Cornell University, Ithaca, NY, United States; ^3^ Department for Horses, Faculty of Veterinary Medicine, Leipzig University, Leipzig, Germany

**Keywords:** equine asthma, heaves, RAO, flow cytometry, cytology, T cells, macrophage

## Abstract

Equine asthma (EA) is a highly relevant disease, estimated to affect up to 20% of all horses, and compares to human asthma. The pathogenesis of EA is most likely immune-mediated, yet incompletely understood. To study the immune response in the affected lower airways, mixed leukocytes were acquired through bronchoalveolar lavage (BAL) and the cell populations were analyzed on a single-cell basis by flow cytometry (FC). Samples of 38 horses grouped as respiratory healthy or affected by mild to moderate (mEA) or severe EA (sEA) according to their history, clinical signs, and BAL cytology were analyzed. Using FC, BAL cells and PBMC were comprehensively characterized by cell surface markers *ex vivo*. An increased percentage of DH24A^+^ polymorphonuclear cells, and decreased percentages of CD14^+^ macrophages were detected in BAL from horses with sEA compared to healthy horses or horses with mEA, while lymphocyte proportions were similar between all groups. Independently of EA, macrophages in BAL were CD14^+^CD16^+^, which contrasts the majority of CD14^+^CD16^-^ classical monocytes in PBMC. Percentages of CD16-expressing BAL macrophages were reduced in BAL from horses with sEA compared to healthy horses. While PBMC lymphocytes predominantly contain CD4^+^ T cells, B cells and few CD8^+^ T cells, BAL lymphocytes comprised mainly CD8^+^ T cells, fewer CD4^+^ T cells and hardly any B cells. These lymphocyte subsets’ distributions were similar between all groups. After PMA/ionomycin stimulation *in vitro*, lymphocyte activation (CD154 and T helper cell cytokine expression) was analyzed in BAL cells of 26 of the horses and group differences were observed (p=0.01–0.11). Compared to healthy horses’ BAL, CD154^+^ lymphocytes from horses with mEA, and CD4^+^IL-17A^+^ lymphocytes from horses with sEA were increased in frequency. Activated CD4^+^ T helper cells were more frequent in asthmatics’ (mEA, sEA) compared to healthy horses’ PBMC lymphocytes. In summary, FC analysis of BAL cells identified increased polymorphonuclear cells frequencies in sEA as established, while macrophage percentages were mildly reduced, and lymphocyte populations remained unaffected by EA. Cytokine production differences of BAL lymphocytes from horses with sEA compared to healthy horses’ cells point towards a functional difference, namely increased local type 3 responses in sEA.

## Introduction

Equine asthma (EA) is a common non-infectious chronic lower airway disease affecting up to 20% of adult horses that impairs welfare and has major economic impact (1–3). EA resembles human asthma in many characteristics, including exacerbation by environmental triggers, and development of bronchospasm, mucus hypersecretion and chronic inflammation (2–4). The equine asthma syndrome is differentiated into phenotypes according to clinical presentation, lung function and airway cytology, best defined in bronchoalveolar lavage fluid (BALF), which is routinely sampled in horses for respiratory diagnostics (1, 3). Mild to moderate asthma (mEA) is differentiated from severe asthma (sEA) by the absence of dyspnea, impaired lung function at rest, and the severity and type of cytologic pathology indicative of airway inflammation. The disease mEA, formerly known as inflammatory airway disease, can have varying cytologic characteristics, i. e. mastocytic, eosinophilic, (mildly) neutrophilic or mixed cytology while sEA (formerly known as heaves or recurrent airway obstruction) is consistently characterized by neutrophilic inflammation (1, 3).

The pathogenesis of EA, in particular the type of underlying immune dysregulation, is still incompletely understood. In contrast to human asthma, pathogenesis-based endotypes of equine asthma have not been defined (3). There are several hypotheses regarding the underlying pathogenesis mechanism of EA spanning from allergic (resembling human type 2 allergic asthma) (3–7), to Th17-mediated (resembling human non-type 2 neutrophilic severe asthma endotypes) (3, 4, 8, 9), or describing dysfunctions of innate mechanisms mediated by macrophages and oxidative stress (2–4, 10).

For the investigation of immune responses in the lung, the analysis of cells from that compartment is most beneficial and has been utilized in various approaches using equine BALF samples (5–7, 9, 11). However, the mixed leukocyte population in BALF and the established differences between healthy and asthmatic horses hamper exact interpretation in many bulk-analysis-based approaches. Accordingly, single-cell-based approaches are more informative and have been performed in more recent studies after the improved availability of new methodology and the development or confirmation of equine-specific antibodies (11–16). Recent reports established several leukocyte phenotype markers for equine BAL cells and PBMC, allowing for in-depth phenotypic characterization of equine leukocytes to elucidate the composition of mixed cell populations (11, 12, 16–18).

We hypothesized that the detailed phenotypes and functions of BAL cells differ between healthy and asthmatic horses. Thus, flow cytometry (FC) was used to comprehensively analyze BAL leukocyte subsets. Furthermore, we compared BAL cells with PBMC as an even more readily available and standardized sample. Importantly, we also analyzed cytokine expression by lymphocytes using intracellular staining of cytokines and FC analysis after *in vitro* stimulation to narrow down a potential T cell polarization bias in EA.

## Material and Methods

### Horses and Diagnostic Evaluation

Horses (n=30) presented as patients for respiratory examination to the Department for Horses, Faculty of Veterinary Medicine, Leipzig University, Germany between March and December 2021 were included in the study after informed consent of their owners. Leftover samples from routine diagnostic procedures were used for research purposes. Additionally, physically healthy control horses (n=8) underwent the same procedures approved under the animal experiment permission number TVV22/20, file number 25-5131/490/23 (Landesdirektion Sachsen, Germany).

The horses’ clinical history was assessed using a questionnaire to score the Horse Owner Assessed Respiratory Signs Index (HOARSI) (19) and a summary HOARSI (range 1–4) was deducted. Information about any treatments in the last month before the examination was recorded. All horses underwent physical examination at rest and after re-breathing, and a clinical score adapted from Ivester et al. (3, 20) was recorded, summarizing nasal discharge, cough, respiratory rate, nasal flare, abdominal lift, and findings on lung auscultation (**Supplementary Table 1**). Arterial blood samples were obtained from the right common carotid artery and analyzed within minutes after collection on an ABL90 FLEX PLUS blood gas analyzer (Radiometer Medical ApS, Brønshøj, Denmark).

Endoscopy of the upper and lower airways was performed after sedation with detomidine and butorphanol with a flexible video endoscope (Storz G28-250, 10.4 mm diameter). A mucus score (21) was assessed by a veterinarian specialized in equine internal medicine. For BALF collection the larynx and the targeted bronchus were anesthetized by topical application of 0.5% lidocaine hydrochloride (bela-pharm, Vechta, Germany) in saline (B. Braun, Melsungen, Germany) *via* the endoscope. The endoscope was advanced into one bronchus until wedged and a lavage was performed using one bolus of saline (60 ml/100 kg body weight, B Braun) with immediate re-aspiration into syringes. The resulting bronchoalveolar lavage fluid (BALF) was placed on ice and processed within one hour after sampling. Heparinized venous blood was acquired by jugular venipuncture (sodium heparin vacuum tubes, Becton Dickinson GmbH, Heidelberg, Germany) and processed within three hours after sampling.

An aliquot of BALF was used to prepare cytospins (5 minutes centrifugation 113 x g, Shandon Cytospin centrifuge, Thermo, Waltham, MA USA); for cytology. These were stained with DiffQuick (RAL Diagnostics, Martillac, France) or toluidine blue (0.25 mg/ml Toluidine Blue O, Sigma-Aldrich, Merck KGaA, Darmstadt Germany). A minimum of 500 cells per specimen was differentiated and the percentages of lymphocytes, macrophages, neutrophil granulocytes (polymorphonuclear cells, PMN), eosinophil granulocytes (all in DiffQuick stained slides), and metachromatic mast cells (MC, in toluidine blue stained slides) were recorded.

### Horse Classification

**Figure 1:**
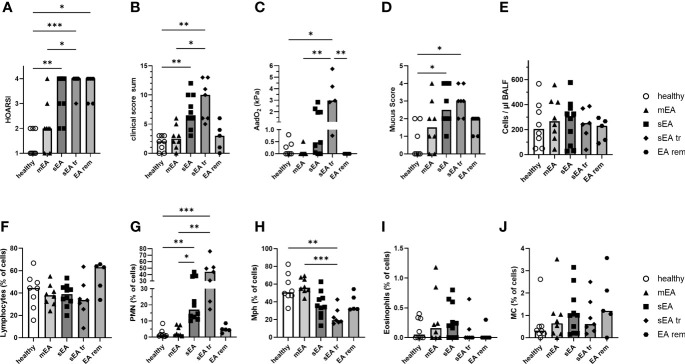
Clinical presentation and BAL cytology confirm group classifications of the horses. Horses were classified as respiratory healthy (n=8) or asthmatic [mild–moderate (mEA, n=8), severe (sEA, n=10), severe and pre-treated (sEA tr, n=7), EA in remission of symptoms (EA rem, n=5)]. The results per group are presented in scatter plots with bars indicating group medians of: **(A)** the history of symptoms (HOARSI), **(B)** clinical presentations (score sum), **(C)** alveolar to arterial oxygen pressure difference (AadO_2_, increased in sEA tr); **(D)** mucus score (increased in sEA and sEA tr) and **(E)** cell counts per µl in BALF. Results from microscopic assessment of nucleated BAL cells (cytospins) are likewise presented for: **(F)** lymphocytes, **(G)** polymorphonuclear cells (PMN), **(H)** macrophages (Mph), **(I)** eosinophils, and **(J)** mast cells (MC). Frequencies of **(G)** PMN were increased in severely asthmatic horses (sEA and sEA tr) and **(H)** frequencies of Mph were reduced in the sEA tr group compared to healthy horses. Asterisks represent differences between groups with p<0.05 in Kruskal-Wallis tests.

The horses were retrospectively classified into groups by established criteria (1, 3) according to their history (Horse Owner Respiratory Signs Index, HOARSI (19)), clinical presentation (**Supplementary Table 1**) (20), endoscopic mucus score (21), BAL cytology, and treatment within four weeks prior to the sampling (**Table 1** and **Figure 1**). Horses with altered complete blood count, fever or other signs of infectious or systemic inflammatory disease were excluded. Horses without a history of asthma symptoms (HOARSI ≦ 2), without findings of lower respiratory disease (dyspnea, cough, mucus nasal discharge, abnormal lung sounds; mucus score ≦2), and with normal BAL cytology (≦ 8% PMN, ≦ 0.5% eosinophils, ≦ 5% MC) were considered respiratory healthy (1, 3). Horses without clinical signs, but abnormal mucus score or BAL cytology were categorized to have mild EA, those with clinical signs but without dyspnea at rest were categorized to have moderate EA. The horses with mild or moderate EA were combined in one group (mEA) according to the consensus statement on inflammatory airway disease (1). Horses with a history of asthma symptoms (HOARSI 3 or 4), clinical findings including dyspnea at rest, a mucus score ≧2, and abnormal BAL cytology with >10% PMN were categorized as horses with severe EA (sEA). Horses in the sEA group who received bronchodilators or corticosteroids within four weeks prior to examination were analyzed as a separate group (sEA tr). Severely asthmatic horses were considered untreated (sEA) if they did not receive any treatment, underwent inhalation of saline, or received mucolytics. Horses with a history of asthma symptoms (HOARSI 3 or 4) but without clinical signs and with mucus scores ≦2 at the time of examination were categorized as EA in remission (EA rem). Horses with BAL samples with indications of blood contamination (macroscopically or microscopically on cytospins) were excluded from the study.

**Table 1:** Horses and group classification.

Group	Total	Healthy	mEA		sEA	sEA tr	EA rem
**Description**		**healthy lungs**	**mild**	**moderate**	**severe (sEA)**	**severe treated (sEA tr)**	**remission**
**HOARSI**		1	1-2	2-3	3-4		3-4
**Clinical score sum**		≤ 3	≤ 3	>3	>5		≤ 3
**Mucus score**^a^		≦2	≦2	>2	>2		≦2
**BAL cytology**		normal	abnormal		abnormal, neutrophilic		normal
**Treatment**^b^		none^c^	none^c^		none^d^	BD or ST^b^	none/ST^e^
**Age**^f^ (years)	13.25 (4–22)	9.5 (3–18)	7* (4–13)		16* (8–22)	14 (4–19)	13 (8–21)
**Sex**^g^	16 m, 2 s, 21 g	5 m, 1 s, 2 g	4 m, 1 s, 3 g		2 m, 8 g	4 m, 3 g	1 m, 4 g
**Type**^h^	3 draft, 6 pony, 6 TB, 24 WB	3 pony, 3 TB, 2 WB	2 TB, 6 WB		2 draft, 2 pony, 6 WB	7 WB	1 draft, 1 TB, 3 WB
**n** (classification, cells *ex vivo*)	38	8	8 (3 mild, 5 moderate)		10	7 (2 BD, 5 ST)	5
**n** (cells stimulated *in vitro*)	26	5	7		6	4	4

a according to Gerber et al. (21);

b treatment within four weeks before sampling: bronchodilators (BD) or corticosteroids (ST);

c one horse received antibiotics prior to sampling;

d two horses received mucolytics;

e one horse received ST;

f median (range); * age difference mEA vs. sEA (p=0.02); all other group comparisons of age were ns;

g m mare, s stallion, g gelding;

h draft horse, pony, TB thoroughbred, WB warmblood.

### Sample Processing

From the same horses used for BAL acquisition, PBMC were isolated from heparinized blood by density gradient centrifugation (Pancoll 1077, Pan-Biotech, Aidenbach) as previously described (13, 15). BALF was passed over a cell strainer (ClearLine100 µm, Kisker Biotech, Steinfurt, Germany) and centrifuged at 500 x g, for 8 min, at 4°C. Supernatant fluid was removed, and the resulting BAL cell pellet was washed in PBS (Roth, Karlsruhe, Germany) with the same centrifugation settings. Viability and counts of PBMC and BAL cells were determined by trypan blue staining and cells were re-suspended in supplemented medium (DMEM low glucose (1 g/l), 1% (v/v) non-essential amino acids (NEA), 2 mM L-glutamine, 50 μg/ml gentamicin, 100 U/ml penicillin, 100 μg/ml streptomycin, 50 μM 2-mercaptoethanol, all from Sigma-Aldrich; 10% heat-inactivated FCS, Lonza, Cologne, Germany) at a concentration of 1x10^7^ live cells/ml. Aliquots of the processed cells *ex vivo* were live/dead stained with fixable viability dye eFluor™506 (eBioscience™, ThermoFisher Scientific) and fixed with 2% Paraformaldehyde in PBS (Roth) or additionally used for *in vitro* stimulation if sufficient BAL cells (2x10^7^ or more live cells) were available from an individual horse. The viability was similar between the cells from healthy and asthmatic horses (BAL cells median 95%, range 80–97%; PBMC median 99%, range 97–100%).

### Stimulation *In Vitro*

BAL cells from 26 horses (5 healthy, 7 mEA, 6 sEA, 4 sEA tr, 4 EA rem) were used for *in vitro* stimulation in comparison to stimulated PBMC from the same horses. *In vitro* stimulation was performed in sterile polystyrene tubes (Sigma) that were pre-incubated with supplemented medium for 3 h at 37°C, aiming to minimize cell adherence *in vitro*. PBMC or BAL cells were incubated in supplemented medium with Brefeldin A (10 µg/ml; Sigma-Aldrich) at 37 °C, 5% CO_2_ in a humidified atmosphere for 4 h. For stimulation a combination of Phorbol-12-myristat-13-acetat (PMA; 25 ng/ml; Sigma-Aldrich) and ionomycin (1 µM; Sigma-Aldrich) was added to the culture medium for the entire incubation time. After 4 h of culture cells were harvested by centrifugation, washed, live/dead stained and fixed as described for the cells used for *ex vivo* phenotyping. Median viability was 95% (range 64-99%) after *in vitro* incubation and stimulation.

### FC Staining and Analysis

The fixed cells were stored at 4°C for a maximum of three days, stained and analyzed as follows: Surface staining was performed in 3% FCS (Lonza), 0.1% NaN_3_ in PBS (Roth), and followed by intracellular staining for *in vitro* cultured cells (CD154, cytokines) in 0.5% Saponin, 3% FCS, and 0.1% NaN_3_ in PBS with different antibody combinations (**Table 2**). 200,000 events per sample were recorded with a BD LSR Fortessa™ Cell Analyzer (BD Biosciences, Ashland, OR, USA) equipped with FACS Diva ™ 6.2 software (BD). Data were analyzed using FlowJo™ v10.8 software (FlowJo, LLC, BD).

**Table 2:** Antibodies and fluorochrome labelling reagents for flow cytometric evaluation.

Antibody - conjugate	Clone	Isotype^d^	Manufacturer	Used in staining panel	Cat. No. or reference
anti-horse CD8-DyL405^a,b,c^	CVS8	msIgG1	Wagner lab, Cornell University, Ithaca, NY, USA	*ex vivo* panel 1, 2 *in vitro* panel 1, 2	(17, 22)
anti-horse CD4-DyL488^a,c^ anti-horse CD4-bio^b,c^	HB61A	msIgG1	Washington State University Monoclonal Antibody Center (WSU)	*ex vivo* panel 1, 2, supplementary experiments *in vitro* panel 1, 2, 3	(13, 15, 22)
anti-horse CD14-bio^a,b,c^	105	msIgG1	Wagner lab	*ex vivo* panel 1, 2, 3 *in vitro* panel 1, 2, 3	(17, 18, 23)
anti-horse CD16-AF647^a,c^	9G5	msIgG1	Wagner lab	*ex vivo* panel 3	(12)
anti-human CD79a-PE^a^	clone HM47	msIgG1	Biolegend	supplementary experiments	(24)
anti-human CD154-APC^b^	5C8	msIgG2a	Miltenyi	*in vitro* panel 1	130-092-290 (25)
anti-horse neutrophil granulocyte^a,f^	DH24A	msIgM	WSU	*ex vivo* panel 1, 3	(11, 16)
anti-horse MHCII-AF647^a,c^	cz11	msIgG1	Wagner lab	*ex vivo* panel 1	(22, 23)
anti-horse IFN-γ-DyL488^b,c^	38-1	msIgG1	Wagner lab	*in vitro* panel 1, 2	(13)
anti-horse IgG (H+L)-Cy3^b,g^	polyclonal	goat	JacksonImmuno Research	*ex vivo* panel 2 *in vitro* panel 2	108-165-003
anti-horse IgE-AF647^a,c^	176	msIgG1	Wagner lab	*ex vivo* panel 2	(23, 26, 27)
anti-horse IL-4-AF647^b,c^	13G7	msIgG1	Wagner lab	*in vitro* panel 3	(28)
anti-horse IL-10-AF647^b,c^	165-1	msIgG1	Wagner lab	*in vitro* panel 2	(29)
anti-horse IL-17A-DyL488^b,c^	76	msIgG1	Wagner lab	*in vitro* panel 3	(8, 14)
anti-horse TNF-α-DyL550^b,c^	48-4	msIgG1	Wagner lab	*in vitro* panel 1	(15)
Iso-DyL405^a,c^	321-2	msIgG1	Wagner lab	*ex vivo* isotype control	
Iso-AF647^a,c^	158-1	msIgG1	Wagner lab	*ex vivo* isotype control	
**Secondary antibody - conjugate**					
anti-mouse IgM-PE^a^	polyclonal	goat F(ab)_2_	JacksonImmuno Research	*ex vivo* panel 1 *ex vivo* isotype control	15-116-075
anti-mouse IgM-Cy2^a^	polyclonal	goat	JacksonImmuno Research	*ex vivo* panel 3	115-225-075
Streptavidin-PE-Cy7^a,b^			BioLegend	*ex vivo* panel 1, 2, 3 *in vitro* panel 1, 2, 3 *ex vivo* isotype control	405206
**Antibody conjugation reagents** ^c^	**NHS ester**				
DyL405	DyLight405		Thermo		46400
DyL488	DyLight405		Thermo		46402
DyL550	Dye 550		Serva		59005.01
AF647	AlexaFluor 647		Thermo		A20006

a used for ex vivo analysis;

b used for in vitro analysis,

c conjugated in-house according to the manufacturer’s instructions,

d ms mouse,

f the DH24A antibody binds CD90 in dogs, but it is unclear if CD90 is also the target on equine granulocytes labelled by DH24A (11, 16),

g the polyclonal anti-horse IgG (H+L) antibody binds all equine immunoglobulin isotypes by the heavy (H) or light chains (L); Staining with this antibody is labelled ‘Ig’ in the results. DyL: DyLight™, AF: AlexaFluor™.

**Figure 2:**
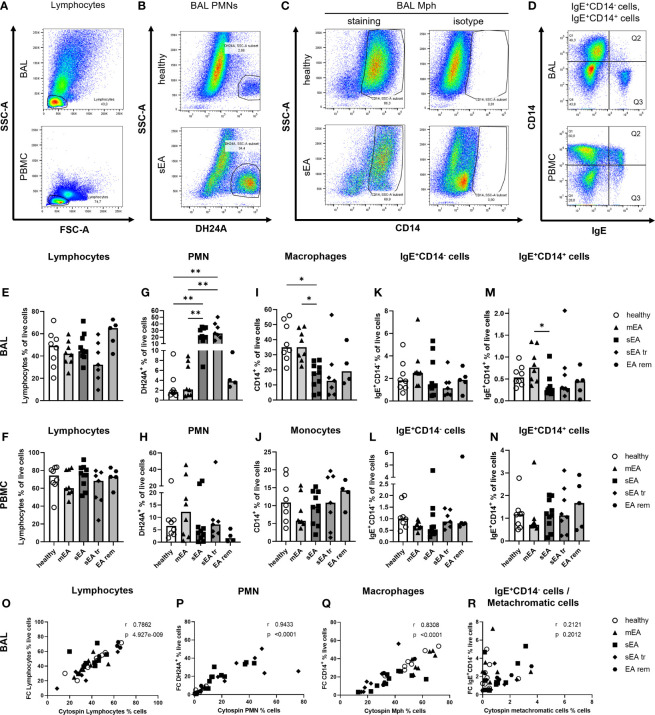
Flow cytometry analysis identifies all major cell populations and confirms increased PMN frequencies in BAL from horses with severe equine asthma (sEA). Representative pseudocolor plots **(A–D)** indicate the gating strategy in different samples (**A, D**: BAL cells or PBMC, sEA) or illustrate differences between groups (**B, C**; healthy or sEA, BAL cells). Singlet live cells were gated as shown in **Supplementary Figure 2** and **(A)** Lymphocytes were gated by FSC-SSC characteristics. After their exclusion, the other cell populations were gated from the non-lymphocyte fraction: **(B)** PMN were identified by staining with antibody clone DH24A, and **(C)** macrophages (Mph)/monocytes by CD14 expression. **(D)** CD14^-^IgE^+^ (Q3), and **(D)** CD14^+^IgE^+^ (Q2) cells were quantified. **(E–N)**. Frequencies of the main cell populations in BAL cells or PBMC were calculated as fractions of all live cells and are plotted by groups. Results from individual horses are shown, with bars indicating median group values. Asterisks represent differences between groups with p<0.05 in Kruskal-Wallis tests. Lymphocyte percentages were similar in **(E)** BAL, or **(F)** PBMC of horses from all groups. Frequencies of **(G)** PMN in BAL of horses with sEA and sEA tr (pre-treated horses with sEA) were higher compared to healthy horses. In contrast, CD14^+^ Mph frequency was decreased in **(I)** BAL from horses with sEA. Percentage of IgE^+^CD14^-^ cells among live cells did not differ between groups in **(K)** BAL and **(L)** PBMC. Proportions of IgE-binding CD14^+^ cells (IgE^+^CD14^+^) were increased in **(M)** BAL cells from horses with mEA compared to the sEA group. The frequencies of the cell populations in PBMC were similar between all groups. EA rem (equine asthma in remission). **(O–R)** Comparisons of cytology differentiation (Cytospin) and flow cytometric (FC) analysis reveal significant correlation of percentages of **(O)** lymphocytes, **(P)** PMN, **(Q)** Mph, but not **(R)** metachromatic cells (MC) and IgE^+^CD14^-^ cells. Spearman r and p are given.

After doublet exclusion by forward scatter (FSC) height against FSC area characteristics (singlets), live cells were selected by exclusion of viability dye positive cells. Then, lymphocytes were gated by FSC and side scatter (SSC) characteristics (**Figure 2**) and lymphocyte and non-lymphocyte (NL) populations were analyzed separately. For *ex vivo* analysis, lymphocyte proportions of CD4^+^, CD8^+^, Ig^+^, and CD79a^+^ cells were quantified. Percentages of CD14^+^, CD16^+^, DH24A^+^, MHCII^high^, and IgE^+^ cells were analyzed in the NL population. For analysis of *in vitro* cultured cells, proportions of CD154^+^, TNF-α^+^, IFN-γ^+^, IL-4^+^, IL-10^+^, and IL-17A^+^ lymphocytes as well as CD4^+^cytokine^+^, and CD8^+^cytokine^+^ double-positive cells were quantified and medium expression percentages were subtracted from those after PMA and ionomycin stimulation resulting in (corrected) net percentages.

### Statistical Analysis

Statistical analysis was carried out using GraphPad PRISM v9 software (GraphPad Software, La Jolla, CA, USA). Non-parametric Kruskal-Wallis test was used for all analyses, as data did not show a Gaussian distribution, followed by Dunn’s Test for multiple comparisons. For the data after *in vitro* stimulation only groups with a minimum of five horses were analyzed statistically (healthy, mEA, sEA). Spearman’s rank correlation was used for assessment of rank correlation between results from cytospin microscopy and FC. Statistical significance was annotated as *p ≤ 0.05, **p ≤ 0.01, ***p ≤ 0.001, ****p ≤ 0.0001.

## Results

### Clinical Disease Classification and Cytospin Analysis of BAL Cells

Cells from bronchoalveolar lavage fluid (BAL cells) and PBMC from 38 horses were isolated for detailed analysis. The horses were retrospectively classified into five groups, based on their history (HOARSI (19) **Figure 1A**), previous treatment with steroids or bronchodilators (sEA tr), clinical presentation (score sum, **Figure 1B**), endoscopic mucus score (21) (**Figure 1D**), and BAL cytology (**Figures 1F–J**). Consequently, HOARSI and clinical score were higher in horses with sEA compared to healthy horses and horses with mEA (**Figures 1A, B**). Alveolar to arterial oxygen pressure difference (AadO_2_) was increased in the sEA tr group (**Figure 1C**). Mucus scores were also higher in horses with sEA and sEA tr compared to healthy horses (**Figure 1D**). The nucleated cell counts per ml were similar in BALF from all groups (**Figure 1E**). BAL cytology, microscopically assessed on cytospins revealed similar frequencies of lymphocytes (**Figure 1F**), eosinophils (**Figure 1I**) and mast cells (MC, **Figure 1J**) in all groups. According to the classification of the horses which included BALF cytology, the percentage of PMN was increased in BAL cells from treated and untreated horses with sEA compared to the other groups (**Figure 1G**). The percentages of macrophages (Mph) among all cells were decreased in the sEA tr group compared to healthy horses and horses with mEA (**Figure 1H**). The increase of PMN percentages in BAL from horses with sEA was also reflected in PMN counts per ml BALF (**Supplementary Figures 1B, G**), while Mph counts per ml BALF (**Supplementary Figures 1C, H, I**) were not significantly different between the groups.

### Flow Cytometric Differentiation Confirms Increased Frequencies of PMN and Decreased Frequencies of Mph in BALF, But Not PBMC From Horses with sEA

Flow cytometry (FC) was used to further characterize BAL cells in comparison to peripheral blood mononuclear cells (PBMC). The percentages of lymphocytes (determined by forward and side scatter, FSC-SSC, characteristics, **Figures 2A, E, F**; detailed gating strategies in **Supplementary Figure 2**), PMN (DH24A^+^, **Figures 2B, G, H**), Mph or monocytes (CD14^+^, **Figures 2C, I, J**), IgE^+^CD14^-^ cells, containing MC (BAL) or basophils, and B cells (PBMC) (**Figures 2D, K, L**), and IgE^+^CD14^+^ IgE-binding Mph/monocytes (**Figures 2D, M, N**) were analyzed.

In BAL cells from horses of all groups the lymphocyte frequencies were similar (overall median 46%, **Figure 2E**), but DH24A^+^ PMN percentages were higher in BAL cells from horses with sEA (median 21%) compared to healthy horses and horses with mEA (both groups median 2%, **Figure 2G**) consistent with results obtained by microscopic cytospin analysis. Flow cytometric PMN identification as DH24A^+^ non-lymphocytes was confirmed by analysis of the MHCII^-^DH24A^+^ non-lymphocyte population, which yielded similar quantitative results (**Supplementary Figure 3**). In further agreement with cytospin analyses, the frequencies of CD14^+^ Mph were lower in BAL cells from horses with sEA (median 18%) compared to healthy horses or the mEA group (both median 35%, **Figure 2I**). Mph percentages in the sEA tr (median 13%) or EA rem (median 19%) groups were similar to sEA, but the differences to Mph percentages in BAL from healthy or mEA horses were not statistically significant (**Figure 2I**). BAL cells’ IgE^+^CD14^-^ (**Figure 2K**) frequencies were again similar between all groups, but IgE-binding CD14^+^ cells frequencies were increased in BAL cells from horses with mEA (median 0.76%) compared to those with sEA (median 0.25%, **Figure 2M**). Calculation of the cell counts per ml BALF confirmed the significant median increase of DH24A^+^ cells in the sEA tr group, but did not reach statistical significance for horses with sEA compared to other groups (**Supplementary Figure 1G**). The calculated counts of other leukocyte subsets per ml BALF were not significantly different between groups (**Supplementary Figures 1F, H–J**). PBMC frequencies of lymphocytes (**Figure 2F**), retained DH24A^+^ PMN (**Figure 2H**), CD14^+^ monocytes (**Figure 2J**), IgE^+^CD14^-^ cells (**Figure 2L**), and IgE^+^CD14^+^ cells (**Figure 2N**) did not differ between the groups.

Results from FC and cytospin analysis correlated for lymphocyte (**Figure 2O**), PMN (**Figure 2P**), and Mph (**Figure 2Q**) frequencies. However, frequencies of IgE^+^CD14^-^ putative MC determined by FC and metachromatic cells in the cytospin analysis did not correlate (**Figure 2R**). For further analysis, we sorted IgE^+^CD14^-^ cells from the DH24A-negative non-lymphocyte fraction by FACS and analyzed their morphology microscopically on cytospins after toluidine blue staining (n=3, healthy, **Supplementary Figure 4**). Microscopic cytospin analysis confirmed major enrichment of metachromatic cells in the sorted IgE^+^CD14^-^ fraction but revealed variable degrees of metachromatic granules per cell (**Supplementary Figure 4I**). Re-analysis with additional staining confirmed the IgE^+^CD14^-^ BAL cells to be MHCII^lo^, consistent with MC (**Supplementary Figure 4G**). Discrepancy of the results obtained by FC and cytospin analysis could result from underestimating the frequency of MC during microscopic assessment of cytospins due to weak metachromatic staining of paucigranulous cells still identified by FC based on their IgE^+^CD14^-^ phenotype.

Taken together, microscopic cytology and FC analyses identify increased percentage of PMN, both in percentual proportions and cell counts per ml BALF, as the major hallmark of sEA, whereas the percentage of Mph was decreased in BAL cells during sEA, but Mph cell counts per ml BALF were similar between all groups.

### Equine BAL Macrophages Are Characterized by Co-Expression of CD14 and CD16 Independent of Asthmatic Disease

**Figure 3:**
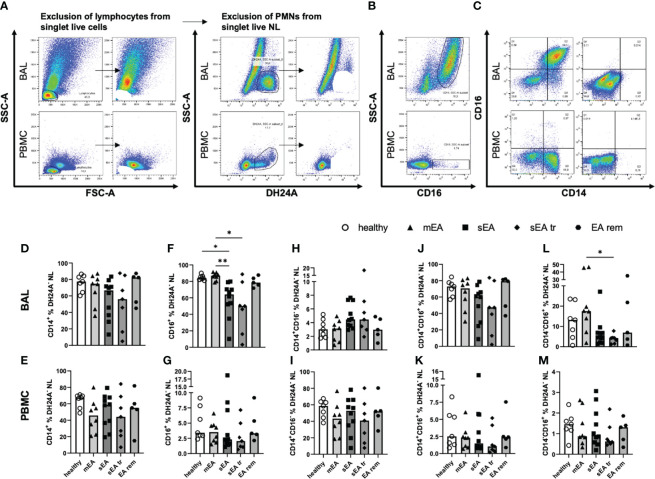
Most BAL macrophages co-express CD14 and CD16. Representative flow cytometry plots of samples from one horse with sEA are shown for gating of **(A)** step-wise exclusion of lymphocytes and PMNs for the further analysis of macrophage (Mph) sub-populations as percentages of DH24A^-^ non-lymphocytes (NL), **(B)** CD16 staining vs. SSC-A, and **(C)** CD14 vs. CD16 gating in BAL (upper row), or PBMC (lower row). The frequencies of CD14^+^ cells among DH24A^-^ NL were similar in samples from horses of all groups in **(D)** BAL cells, or **(E)** PBMC. In contrast, the frequencies of CD16^+^ cells of DH24A^-^ NL were lower in **(F)** BAL from horses with sEA compared to healthy horses, but not different between groups in **(G)** PBMC. Analysis of CD16 vs. CD14 expression on BAL cells **(H, J, L)** and PBMC **(I, K, M)** revealed similar distribution of CD14 and CD16 expression on macrophages and monocytes between the groups, except **(L)** a decreased percentage of CD14^-^CD16^+^ cells in BAL samples of pre-treated sEA horses (sEA tr) compared to horses with mild to moderate EA (mEA). Results of individual horses (n=5–10 per group) are shown with bars indicating median values and asterisks indicating differences between groups with p<0.05 in Kruskal-Wallis tests. EA rem (equine asthma in remission).

The phenotypes of BAL Mph and PBMC monocytes were further characterized according to their expression of the classical monocyte/Mph marker CD14 (lipopolysaccharide co-receptor (17, 18),), and CD16 (FcγRIIIa), which has been described and used as a non-classical monocyte marker (12, 17). Staining of mannose receptor CD206 and scavenger receptor CD163, other markers of equine BAL Mph (11, 30), was performed on some BAL samples and the majority of the putative Mph (70-90% of DH24A^-^ NL) were positive for both, CD14 and CD206 or CD14 and CD163 (data not shown). Due to less interference with Mph autofluorescence in FITC and PE (compare (11)) with the available conjugates for CD14 antibodies compared to CD206 or CD163, and the indication of regulation of the latter two surface molecules (11), we selected CD14 as the main marker for Mph identification and quantification in our analyses (compare **Figures 2C, E**). To correct for the increased PMN frequencies in BAL from horses with sEA, Mph were gated from non-lymphocytes after PMN exclusion (**Figure 3A**) and quantified as percentage of DH24A-negative non-lymphocytes (DH24A^-^ NL, detailed gating strategy in **Supplementary Figure 5**). The DH24A^-^ NL population contains mainly Mph, but may include small fractions of other cells, such as MC and eosinophils, which are neither excluded by lymphocyte nor by DH24A (PMN) gating.

The majority of BAL Mph co-expressed CD14 and CD16 (**Figures 3C, H**), while the main population of PBMC monocytes were CD14^+^CD16^-^ cells (**Figures 3C, K**). The frequencies of CD14^+^ Mph from horses of different groups, were not significantly different in DH24A^-^ NL in BAL (**Figure 3D**) or PBMC (**Figure 3E**) in contrast to the frequencies obtained relative to all singlet live cells (**Figures 2I, J**, without correction for the PMN). However, the frequencies of CD16^+^ cells (**Figure 3B**) were still reduced in BAL DH24A^-^ NL from horses with sEA (median 64%) compared to healthy horses (median 84%) and to the mEA group (median 87%, **Figure 3F**). Similarly, frequencies of CD14^-^CD16^+^ cells were lower in the sEA tr group compared to the mEA group among DH24A^-^ NL BAL cells (**Figure 3L**). Nevertheless, the percentages of CD14^+^CD16^-^ cells (**Figure 3H**) and the dominating CD14^+^CD16^+^ (**Figure 3J**) cells were similar in BAL samples of all groups. The horses’ PBMC had similar frequencies of CD14^+^ and CD16^+^ monocytes in samples of all groups (**Figures 3E, G, I, K, M**). In summary, CD16^+^ Mph may be mildly reduced within BAL Mph from horses with sEA even if the main population of BAL Mph (CD14^+^CD16^+^) appears unaffected by the disease.

**Figure 4:**
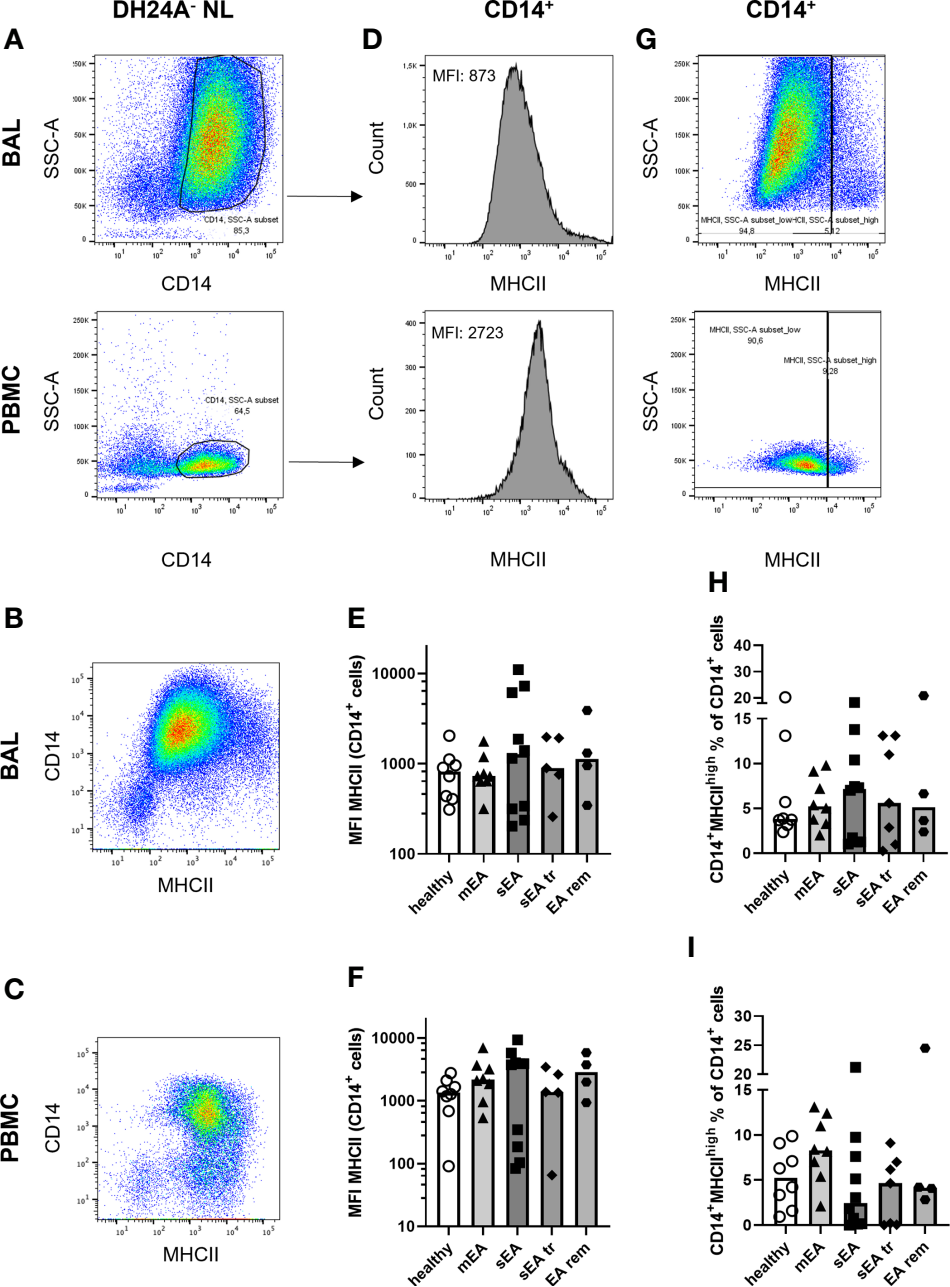
MHCII expression on CD14^+^ macrophages is similar in BAL samples from horses of all groups. Representative flow cytometry plots of one healthy horse’s BAL cells (upper row) and PBMC (lower row) are shown for **(A)** singlet live DH24A^-^ non-lymphocytes (NL) gated for CD14^+^ cells. MHCII vs. CD14 in DH24A^-^ NL is shown for comparison in **(B)** BAL cells, **(C)** PBMC. MHCII expression on CD14^+^ cells **(A)** was quantified as **(D)** median fluorescence intensity (MFI), and **(G)** percentage of MHCII^high^ cells. MHCII expression (MFI) on CD14^+^ DH24A^-^ NL was similar on **(E)** BAL cells, or **(F)** PBMC and frequencies of MHCII^high^CD14^+^ cells in **(H)** BAL cells, or **(I)** PBMC were similar between horses from all groups. mEA (mild to moderate equine asthma), sEA (severe equine asthma), tr (pre-treated), EA rem (equine asthma in remission).

Additionally, MHCII expression (marking professional antigen-presenting cells (17)) on CD14^+^ Mph was quantified to analyze potential MHCII regulation in healthy or asthmatic airway leukocytes (**Figure 4**). MHCII expression was similar on CD14^+^ BAL Mph (MHCII median fluorescence intensities, **Figures 4D, E**) or PBMC monocytes (**Figure 4F**) of all groups ranging from MHCII^lo^ to MHCII^hi^ CD14^+^ cells (**Figures 4B, C**). Quantification of a small, distinct population of CD14^+^ MHCII^high^ cells (**Figure 4G**) also resulted in similar frequencies in BAL or PBMC samples from horses of all groups (overall median 5% of CD14^+^ NL, **Figures 4H, I**).

In summary, we newly identify CD14^+^CD16^+^ cells as the largest sub-population among Mph in BAL which is unaffected by EA, and demonstrate reduction of CD14^-^CD16^+^ BAL cells in sEA, whereas PBMC monocytes are unaffected by EA and are mostly CD14^+^CD16^-^, as previously established in horses (12, 17).

### Cytotoxic T Cells Dominate the Lymphocytes in BAL Cells of Healthy and Asthmatic Horses in Contrast to Their PBMC

**Figure 5:**
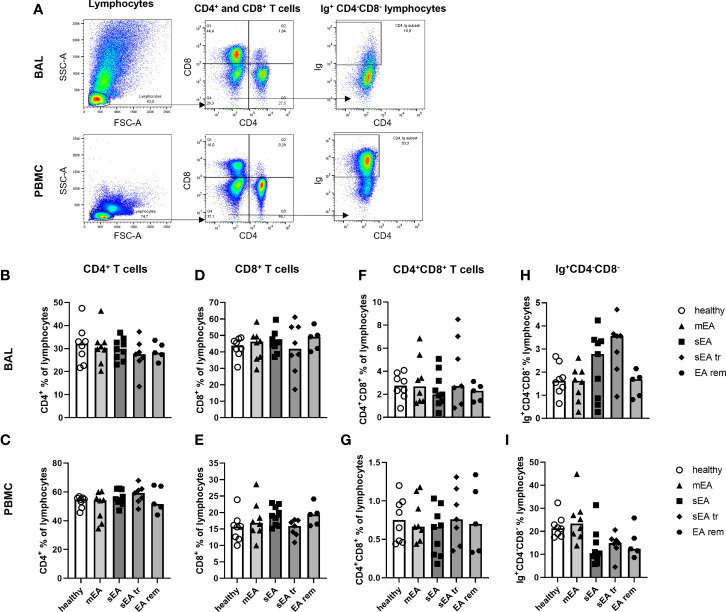
Proportions of CD4^+^ T cells, CD8^+^ T cells, and Ig^+^ lymphocytes are different in BAL cells and PBMC, but similar between groups. **(A)** Representative flow cytometry plots for gating of lymphocyte populations are depicted for samples from one horse with severe equine asthma (sEA). CD4^+^ T cells **(B, C)**, CD8^+^ T cells **(D, E)**, CD4^+^CD8^+^ T cells **(F, G)**, as well as Ig^+^CD4^-^CD8^-^ lymphocytes **(H, I)** were detected at similar frequencies among BAL cells or PBMC from horses of all groups. Values of individual horses are shown with bars indicating group medians. Mind the different scales of the Y-axes. mEA (mild to moderate equine asthma), sEA (severe equine asthma), tr (pre-treated with steroids or bronchodilators), EA rem (equine asthma in remission).

The lymphocyte sub-populations of equine BAL cells and PBMC were analyzed by FC (**Figure 5A**). The staining with CD8 was not valid for samples from one horse (sEA group), which was thus excluded from the lymphocyte subset analysis. The proportions of CD4^+^ T helper cells (**Figures 5B, C**), CD8^+^ cytotoxic T cells (**Figure 5D, E**), CD4^+^CD8^+^ T cells (**Figures 5F, G**), and Ig^+^CD4^-^CD8^-^ cells (**Figures 5H, I**) among lymphocytes were similar in BAL cells or PBMC between the different groups of horses. However, CD8^+^ cytotoxic T cells dominated the lymphocytes in BAL cells (overall median 55% of lymphocytes, **Figure 5D**) independent of asthmatic disease, in contrast to PBMC samples containing mainly CD4^+^ T helper cells (median 46% of lymphocytes, **Figure 1C**). Ig^+^CD4^-^CD8^-^ cells were very rare in BAL lymphocytes (median 2%) in contrast to PBMC lymphocytes (median 18%, **Figure 5H, I**). Staining of CD79a was applied to further characterize the Ig^+^CD4^-^CD8^-^ lymphocytes (**Supplementary Figure 6**). In PBMC, Ig and CD79a were co-expressed on most CD4^-^CD8^-^ lymphocytes (**Supplementary Figure 6G**), confirming them as B cells. However, in BAL cells only few Ig^+^CD4^-^CD8^-^ lymphocytes expressed CD79a (**Supplementary Figure 6D**) and the percentages of Ig^+^CD4^-^CD8^-^ and CD79a^+^CD4^-^CD8^-^ lymphocytes did not correspond in individual horses (**Supplementary Figure 6E**). Thus, the small fraction of Ig^+^CD4^-^CD8^-^ lymphocytes found in BAL can only partially be considered B cells and the roughly 20% CD4^-^CD8^-^ lymphocytes in BAL are different from typical B or T cells. Those cells could represent non-conventional T cells, natural killer cells, or innate lymphoid cells, which could not be differentiated further with the markers available here.

### *In Vitro* Stimulated Lymphocytes Reveal a Trend of Increased Th17 Cells in BAL From Severely Asthmatic Horses

**Figure 6:**
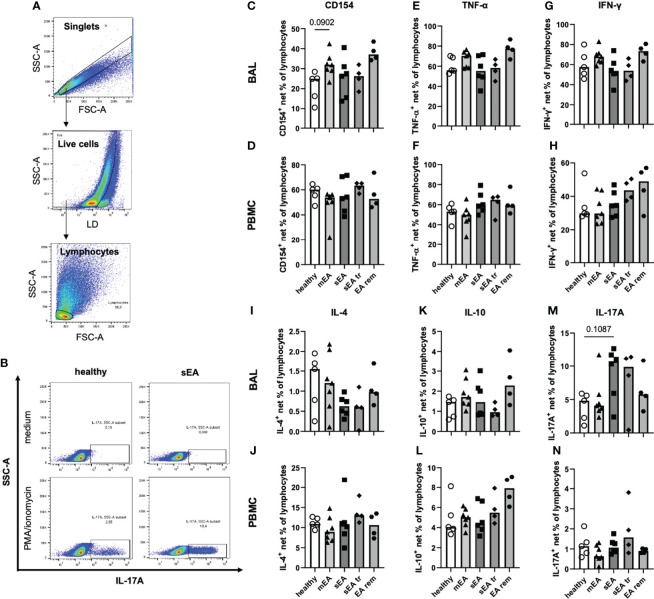
*In vitro* stimulation of equine BAL lymphocytes induces expression of several cytokines with a bias toward increased IL-17A expression in sEA. Representative plots show the gating of **(A)** singlet live lymphocytes in BAL from one horse with sEA. **(B)** Representative plots of cytokine-expressing BAL lymphocytes (IL-17A) of one healthy and one asthmatic (sEA) horse after *in vitro* incubation in medium alone or stimulation with PMA/ionomycin are shown. Medium-corrected net percentages of CD154- or cytokine-expressing lymphocytes are displayed for BAL cells **(C, E, G, I, K, M)** and PBMC **(D, F, H, J, L, N)**. Results of individual horses (total n=26) are shown with bars indicating median values (n=4–7 per group). Expression of CD154 and cytokines was not significantly different in BAL cells or PBMC between horses from different groups. Trends are indicated by given p-values (p<0.12 in Kruskal-Wallis tests comparing groups with n≥5: healthy, mEA, sEA). In BAL lymphocytes there were trends of **(C)** higher CD154^+^ frequencies in BAL lymphocytes from horses with mEA, and **M**) higher IL-17A^+^ frequencies in BAL lymphocytes from sEA, compared to healthy horses.

Beyond demonstrating similar frequencies of T helper cells, cytotoxic T cells and B cells in samples from all groups *ex vivo*, we aimed to functionally characterize these lymphocytes by analysis of the T helper cell activation marker CD154 (CD40L) (25, 31) and expression of five different cytokines after *in vitro* stimulation with PMA and ionomycin (**Figures 6A, B**). *In vitro* stimulation was performed for samples from 26 horses (n=4–7 per group) with sufficient yield of BAL cells, and only groups with five or more values (healthy, mEA, sEA) were compared statistically. Net percent of cytokine-expressing lymphocytes of all live lymphocytes were analyzed after medium correction (**Figures 6A, B**).

The frequencies of CD154-expressing lymphocytes were similar in BAL cells of horses from all groups, except for a trend towards higher frequencies in horses with mEA (p=0.09) compared to healthy horses (**Figure 6C**) and elevated median percentages in the EA rem group (not statistically analyzed, **Figure 6C**). The proportions of lymphocytes expressing TNF-α (**Figure 6E**), IFN-γ (**Figure 6G**), IL-4 (**Figure 6I**), or IL-10 (**Figure 6K**) were similar in BAL cells from horses of all groups. However, IL-17A^+^ lymphocytes were present at higher frequencies among BAL cells from horses with sEA compared to the other groups, although this trend did not reach statistical significance (p=0.11 sEA median 10.7% *vs*. healthy median 4.8%, **Figure 6M**). Similar percentages of CD154 and cytokine-expressing lymphocytes were detected in PBMC lymphocytes of horses from all groups (**Figures 6D, F, H, J, L, N**).

Interestingly, TNF-α was similarly induced in BAL and PBMC lymphocytes (overall medians, **Figures 6E, F**), but higher frequencies of IFN-γ^+^ (2-fold, **Figure 6G**), and IL-17A^+^ cells (6-fold, **Figure 6M**) were detected in BAL lymphocytes compared to PBMC lymphocytes (**Figures 6F, H, N**), whereas lower CD154^+^ (2-fold, **Figure 6C**), IL 4^+^ (13-fold, **Figure 6I**), and IL-10^+^ (4-fold, **Figure 6K**) BAL lymphocyte frequencies were detected compared to PBMC lymphocytes (**Figures 6D, J, L**).

**Figure 7:**
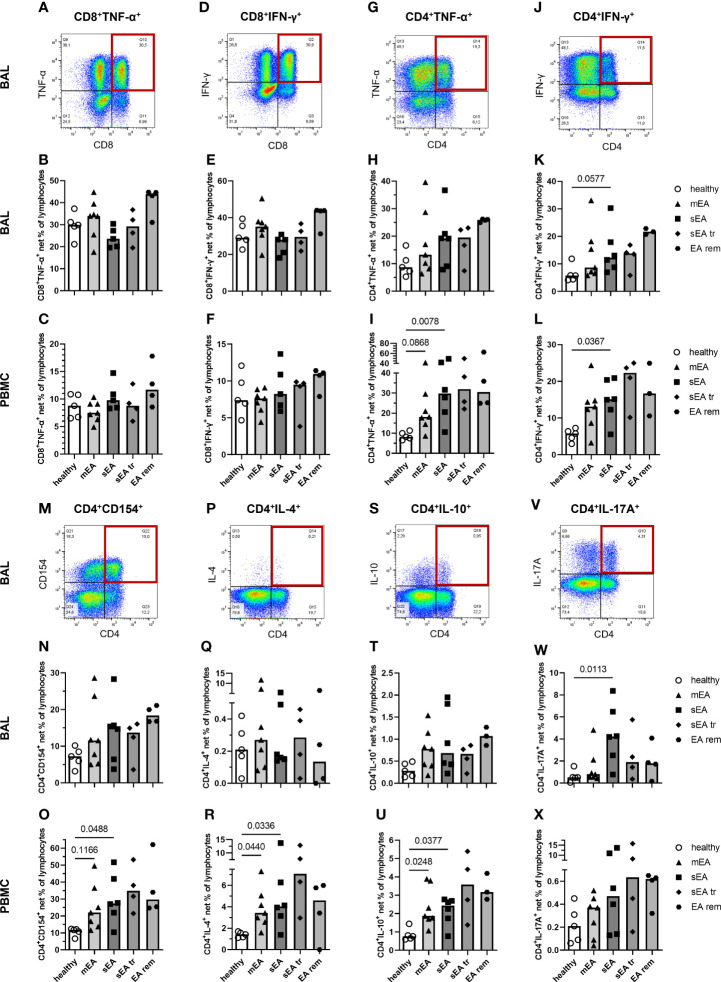
*In vitro* stimulated T cells indicate a local Th17 bias during severe equine asthma and broad Th cell activation in asthmatic horses’ PBMC. Representative plots with quadrant gates on singlet live lymphocytes after stimulation with PMA and ionomycin are shown for BAL from one horse with severe equine asthma (sEA) **(A, D, G, J, M, P, S, V)**. CD8^+^, or CD4^+^ lymphocytes expressing TNF-α, or IFN-γ, and CD4^+^ lymphocytes expressing CD154, IL-4, IL-10, or IL-17A are displayed as medium-corrected net percentages in BAL **(B, E, H, K, N, Q, T, W)** and PBMC lymphocytes **(C, F, I, L, O, R, U, X)**. Mind the variable scales of the y-axes. Results of individual horses (total n=26) are shown with bars indicating median values (n=4–7 per group). Differences between groups of n≥5 (healthy, mEA, sEA) with p<0.12 in Kruskal-Wallis tests are indicated by given p-values. The frequencies of CD4^+^IFN-γ^+^ Th1 cells (p=0.058, **K**) and CD4^+^IL-17A^+^ Th17 cells (p=0.011, **W**) were higher in BAL lymphocytes from horses with sEA than from healthy horses. In PBMC, net percentages of activated Th cells were increased in sEA and mEA lymphocytes compared to healthy horses’ cells [**I**( CD4^+^TNF-α^+^, **L**) CD4^+^IFN-γ^+^, **(O)** CD4^+^CD154^+^, **(R)** CD4^+^IL-4^+^, and **(U)** CD4^+^IL-10^+^ cells].

We further characterized the T cells as potent activated cells and cytokine producers in the lymphocyte population through staining of CD4 and CD8 combined with staining of intracellular cytokines and CD154 (**Figure 7**). CD4^+^ and CD8^+^ T cells expressed TNF-α and IFN-γ in BAL and PBMC lymphocytes (**Figures 7A–L**). CD8^+^TNF-α^+^ and CD8^+^IFN-γ^+^ cytotoxic T cells (CTL) were stimulated at 3-fold higher frequencies in BAL cells compared to PBMC (**Figures 7A–F**), matching the results from *ex vivo* analysis with higher frequencies of CD8^+^ T cells in BAL cells compared to PBMC (**Figures 5D, E**). The percentages of CD8^+^TNF-α^+^ and CD8^+^IFN-γ^+^ CTL frequencies were similar in all groups’ BAL cells or PBMC (**Figures 7B, C, E, F**).

Frequencies of putative T helper (Th) 1 cells, defined as CD4^+^TNF-α^+^ (**Figure 7G**) and CD4^+^IFN-γ^+^ (**Figure 7J**) cells, were higher in BAL of horses with sEA compared to healthy horses’ BAL lymphocytes (**Figures 7H, K**), but this only tended toward statistical significance for CD4^+^IFN-γ^+^ percentages (p=0.058, **Figure 7K**). Frequencies of CD4^+^CD154^+^ (activated Th cells, **Figures 7M-O**), CD4^+^IL-4^+^ (Th2, **Figures 7P–R**), and CD4^+^IL-10^+^ (T_reg_, **Figures 7S–U**) frequencies were lower in BAL cells than PBMC but were similar in BAL cells of all groups. The trend of increased CD154^+^ lymphocytes in mEA BAL (**Figure 6C**) was not resembled in BAL Th CD4^+^CD154^+^ cells (ns, **Figure 7N**). In agreement with all IL-17A^+^ lymphocytes (**Figure 6M**) however, the frequencies of CD4^+^IL-17A^+^ lymphocytes (Th17, **Figure 7V**) were higher in BAL cells than in PBMC and were significantly increased in BAL lymphocytes from horses with sEA compared to healthy horses (p=0.011, **Figures 7W, X**).

In PBMC, the median frequencies of activated Th cells expressing cytokines or CD154 after stimulation were higher in asthmatic - than in healthy horses’ lymphocytes (sEA>mEA>healthy, **Figures 7I, L, O, R, U, X**) in contrast to all lymphocytes’ cytokine expression without such clear group differences (**Figure 6**). Nevertheless, the frequencies of activated Th cells were not statistically different between horses with mEA and sEA, but significantly higher in PBMC lymphocytes from horses with sEA than healthy horses’ PBMC lymphocytes for CD4^+^TNF-α^+^ (**Figure 7I**), CD4^+^IFN-γ^+^ (**Figure 7L**), CD4^+^CD154^+^ (**Figure 7O**), CD4^+^IL-4^+^ (**Figure 7R**), and CD4^+^IL-10^+^ (**Figure 7U**) cells. Proportions of activated Th cells were significantly higher in PBMC of horses with mEA compared with healthy horses for CD4^+^IL-4^+^ (**Figure 7R**) and CD4^+^IL-10^+^ (**Figure 7U**) cells and had a similar trend for CD4^+^TNF-α^+^ (**Figure 7I**) and CD4^+^CD154^+^ (**Figure 7O**) lymphocytes. CD4^+^IL-17A^+^ cells showed a similar expression distribution in PBMC like the other Th cells, but the increased medians of CD4^+^IL-17A^+^ in asthmatic horses’ PBMC did not reach a statistically significant difference compared to healthy horses’ PBMC (**Figure 7X**).

Co-expression of CD154 and the Th subset differentiating cytokines IFN-γ, IL-4, and IL17A was usually observed in BAL cells and PBMC, but was incomplete, i. e. not all cytokine expressing Th cells simultaneously expressed CD154 (**Supplementary Figures 7A–C**), as previously described for equine PBMC (25). The frequencies of CD154^+^ lymphocytes co-expressing different cytokines in BAL and PBMC samples (**Supplementary Figures 7A–C**) showed distributions between the groups comparable to all cytokine-positive lymphocytes (**Figure 6**).

Cytokine and CD154 expression were induced in CD4^+^ Th cells in BAL cells and PBMC, but also in CD4^-^ lymphocytes (**Figures 7A, D, G, J, M, P, S, V**). Additional experiments with cells from three healthy horses (**Supplementary Figures 7D–H**) confirmed that the cytokine producing lymphocytes are mainly composed of CD4^+^ or CD8^+^ T cells, but CD4^-^CD8^-^ lymphocytes also contribute to cytokine expression, particularly to IL-17A expression in BAL lymphocytes (**Supplementary Figure 7H**).

## Discussion

The results of this study provide extensive insight into the cellular composition and functional features of equine BAL cells compared to PBMC. We classified horses as healthy or asthmatic according to established criteria minding the phenotypical subtypes of the equine asthma syndrome defined by clinical and cytologic criteria (1, 3). For in-depth analysis of BAL and PBMC samples, we used a state-of-the-art multicolor FC approach, confirmed and validated by standard microscopic assessment of cytospins. Data from FC and cytospin analysis correlated strongly for the main populations, i. e. PMN, Mph, and lymphocytes.

An increased frequency of PMN in BAL samples of symptomatic sEA could be determined by FC and microscopy alike, with clear correlation and in agreement with previous studies (5, 7, 11, 30). In single horses’ samples DH24A staining also marked putative CD14^+^ Mph with a high SSC (data not shown). These need to be excluded from PMN quantification, either by SSC, as done here, or by the combination of CD14 vs. DH24A if FC was used for diagnosis of asthma based on PMN quantification without cytospin comparison.

Conversely, frequencies of IgE^+^CD14^-^ using FC and MC identified as metachromatic cells on cytospins in BAL did not correlate. Sorting of putative MC (IgE^+^CD14^-^ cells) by FACS yielded enrichment of metachromatic cells with considerably varying metachromatic granules per cell. Thus, a) the majority of IgE^+^CD14^-^ BAL cells can be considered MC, and b) the frequencies of MC may have been underestimated during microscopic analysis of cytospins, leading to contradictory results between the two methods used for quantification here. However, the percentage of IgE^+^CD14^-^ cells quantified by FC or MC on cytospins was similar in BALF from all groups, which is in agreement with previous studies (2, 5). The IgE^+^CD14^-^ cell fraction in PBMC samples contains basophils and B cells (31, 32) and was unaffected by EA. In addition to IgE^+^CD14^-^ cells, we quantified IgE-binding Mph and monocytes (IgE^+^CD14^+^ cells). IgE-binding monocytes have been suggested to contribute to the pathogenesis or regulation of allergic disease in horses through production of IL-8 (27), or IL-10 (23), respectively. Interestingly, IgE^+^CD14^+^ cells were present at higher frequencies in BAL cells from horses with mEA compared to sEA horses, hinting towards different endotypes of allergic or non-allergic asthma pathogenesis dominating those two phenotypes of EA. Moreover, the lack of elevated eosinophil percentage in BAL cells (3), does not support an allergic pathogenesis in sEA. Similar to previous studies, we also show overall minimal eosinophil percentages in BAL cells from horses of all groups. For mEA a Th2 bias was furthermore not indicated by our analyses.

BAL cell composition of horses with sEA pre-treated with bronchodilators or steroids resembled the results from untreated horses with sEA, despite the more severe clinical presentation of the sEA tr group, as shown by increased AadO_2_. BAL cells from horses with EA rem were not different from those from other groups examined regarding phenotypes and frequencies. The lack of differentiation is likely impacted by the heterogeneity of the EA rem group regarding the underlying disease phenotype when not in remission (mEA or sEA), and the presence or absence of previous treatment.

The overall Mph proportion was reduced in BAL from horses with sEA, similar to previous findings (5, 7, 30). However, Mph cell counts per ml BALF were not significantly different between healthy horses and horses with sEA. Their relative reduction may thus primarily be caused by PMN influx, resulting in a percentual shift, rather than by an absolute reduction of Mph numbers in the lungs. Nevertheless, we characterized the phenotype of Mph in more depth based on their expression of the lipopolysaccharide co-receptor CD14, expressed on most equine monocytes (17, 18) and equine alveolar Mph (33), and expression of FcγRIIIa CD16, expressed only on few equine blood monocytes (12, 17, 23). The two markers were used based on their association with different monocyte subsets, i. e. CD14 is regarded as a marker for classical monocytes and Mph, while CD16 is expressed on equine monocytes exhibiting a gene-expression profile similar to human non-classical monocytes (17). These markers were also applied for differentiation of bovine monocytes into classical (CD14^+^CD16^-^), intermediate (CD14^+^CD16^+^) and non-classical (CD14^-^CD16^+^) monocytes (34) similar to equine monocytes (17, 23). In our analyses co-expression of CD14 and CD16 was observed on most BAL Mph in contrast to blood monocytes. This is in contrast to findings in humans, where CD14^high^CD16^high^ cells only accounted for 1–5% of the bronchial Mph (35). However, the percentages of CD14^+^CD16^+^ BAL Mph did not differ between the groups of horses compared here. In contrast to the consistent CD14^+^CD16^+^ main Mph population, a decreased proportion of CD16-expressing Mph in BAL cells from horses with sEA was observed. Accordingly, CD16^+^ Mph in the lungs may be affected by a dysregulated immune response during sEA. These results agree with those of Moniuszko et al., who found decreased CD16 expression on human bronchial Mph in asthmatic patients compared to healthy individuals, hinting towards a role of CD16 in regulation of the inflammatory response in the lungs (35).

In depth characterization of lymphocytes revealed comparable frequencies of CD4^+^ T helper cells and CD8^+^ cytotoxic T cells in BAL cells, and PBMC from all groups. However, the majority of T cells in equine BAL were CD8^+^, confirming previous studies (36, 37). Conversely, PBMC T cells were mainly CD4^+^ (17, 37). In humans the CD4/CD8 ratio of BAL lymphocytes is normally 0.9–2.5 (38), i. e. CD4 T helper cells usually dominate the T lymphocytes in contrast to our findings in horses. We further confirmed that B cells are either absent or present at minimal frequencies in BALF, as previously shown in other studies in horses (36, 37) and humans (39). Nonetheless, CD4^-^CD8^-^ lymphocytes, which also expressed cytokines, were prominent in BAL and could represent innate lymphoid cells. It has been suggested that these contribute to the pathogenesis in human asthma *via* cytokine secretion (40). A contribution of innate lymphoid cells to equine asthma is discussed, but has not been established (3).

Apart from the composition of BAL cells from healthy and asthmatic horses, functional characterization of lymphocytes was carried out to elucidate mechanisms contributing to the sustained inflammation present in EA. We analyzed lymphocytes’ expression of cytokines, and the T helper cell activation molecule CD154 (25, 41) after stimulation with PMA and ionomycin. Lymphocytes expressing the pro-inflammatory cytokines TNF-α and IFN-γ after stimulation were overall present at similar frequencies in BAL cells from horses of all groups, except for a trend of increased Th1 activation (CD4^+^IFN-γ^+^) in BAL lymphocytes from horses with sEA. This is in contrast to previous studies reporting increased IFN-γ mRNA expression in BAL cells (30), and higher concentration of IFN-γ and TNF-α in BALF from sEA-susceptible compared to healthy horses (42). This discrepancy could result from the separate analysis of lymphocytes or T cells carried out in our study in contrast to the analysis of mixed cell populations or their secretions in previous studies.

In our study, frequencies of CD4^+^IL-4^+^ Th2 cells were similar in BAL of all groups and the median frequencies of IL-4^+^ or CD154^+^IL-4^+^ BAL lymphocytes, indicative of a type 2 bias were similar or lower in the sEA group compared to healthy or mEA without reaching statistical significance. This matches previously demonstrated comparable IL-4 mRNA expression in BAL cells from sEA-susceptible horses and healthy horses (30). This finding contradicts the previous paradigm of a Th2-driven immune response and allergic pathogenesis in sEA, which was supported by studies demonstrating increased percentages of IL-4- and IL-5-expressing, but lower frequencies of IFN-γ-expressing BAL cells in horses with sEA compared to healthy horses using *in situ* hybridization analysis (6, 7).

Of note, the frequencies of IL-17A^+^ and CD4^+^IL-17A^+^ lymphocytes, indicative of Th17 cells, were increased in stimulated BAL cells from horses with sEA compared to healthy horses, suggesting an increased type 3 response in sEA rather than a type 2 response. Furthermore, IL-17A expression in stimulated BAL lymphocytes was higher compared to PBMC even though the frequencies of CD4^+^ Th cells in PBMC exceeded those in BAL cells indicating a strong local bias towards a type 3 response in lungs of horses, particularly those with sEA. An increased type 3 response driving sEA is further supported by studies reporting a higher IL-17 to CD3 mRNA ratio in BAL cells from horses with sEA compared to healthy horses (9), and upregulation of IL-17 signaling pathways and higher protein expression of IL-17 in mediastinal lymph nodes from sEA-susceptible horses compared to healthy horses after challenge with dusty hay (8). However, another study found similar upregulation of IL-17 mRNA expression in BAL cells from both sEA-susceptible and healthy horses in response to challenge with hay dust (43). Increased numbers of IL-17-secreting lymphocytes could contribute to the pathology of sEA, as IL-17 was demonstrated to increase IL-8 mRNA expression and the viability of equine PMN (44), which are a hallmark of the inflammation in sEA.

In contrast to the Th17 bias and a trend of increased Th1 in BAL cells from sEA only, all Th subsets were more strongly stimulated in PBMC of asthmatic, mEA or sEA, horses than in healthy horses’ PBMC. This was statistically significant for the frequencies of CD4^+^CD154^+^, CD4^+^TNF-α^+^, CD4^+^IFN-γ^+^, CD4^+^IL-4^+^, and CD4^+^IL-10^+^ lymphocytes in sEA and this variety may be more reflective of a general activation of Th cells than a bias towards a certain polarization in PBMC. Systemic inflammatory effects and priming of PBMC have been reported before (3, 45), and may particularly affect circulating T helper cells of asthmatic horses. The specific analysis of CD4^+^ Th cells for several cytokines was superior in demonstrating these effects compared to the analysis of the cytokines in all leukocytes or all lymphocytes, which are still a mixed cell population. This argues for detailed analyses like cell subset analyses by FC in the complex pathology of equine asthma.

## Conclusions

We demonstrated that FC is a suitable method for characterization of routinely acquired BAL cells from diagnostic procedures with the potential to elucidate the mechanisms of immune dysregulation at the effector site of equine asthma. This can form the basis for further research to elucidate the pathogenesis and ultimately inform targeted treatment options for equine asthma. Data from our *in vitro* stimulation assays suggest a polarization towards a type 3 immune response rather than a type 2 response in severe equine asthma, based on increased Th17 cell percentages in horses with sEA compared to healthy horses. Additionally, neither percentages of eosinophils, or IgE-binding cells, nor frequencies of IL-4^+^ lymphocytes were increased in horses with sEA, contradicting an excessive type 2 immune response and allergy as the underlying pathogenesis mechanism of sEA. The clear distinction of horses with sEA from those with mEA regarding cell composition and cytokine expression supports different types of pathogeneses in these two phenotypes of equine asthma and points towards mEA as an endotype separate from sEA.

## Data Availability Statement

The raw data supporting the conclusions of this article will be made available by the authors, without undue reservation.

## Ethics Statement

The animal study was reviewed and approved by Landesdirektion Sachsen, Germany. Written informed consent was obtained from the owners for the participation of their animals in this study.

## Author Contributions

CS conceptualized the study and acquired funding. AG and CS designed experiments and drafted the initial manuscript. KL and CA performed clinical exams, endoscopic grading and acquired history information from horse owners. CA, KL, and CS performed BAL endoscopy. AG, SL, and CS processed the samples, performed cell culture, microscopy, and flow cytometry experiments, and analyzed the data. BW developed and provided key reagents and methodological advice. All authors contributed to the article and approved the submitted version.

## Funding

CS, AG, and SL, as well as materials and the experimental horses are funded by the German Research Foundation (DFG), Emmy-Noether-Programme, project number 431342499. The authors further acknowledge support from the German Research Foundation and Universität Leipzig within the program of Open Access Publishing.

## Conflict of Interest

The authors declare that the research was conducted in the absence of any commercial or financial relationships that could be construed as a potential conflict of interest.

## Publisher’s Note

All claims expressed in this article are solely those of the authors and do not necessarily represent those of their affiliated organizations, or those of the publisher, the editors and the reviewers. Any product that may be evaluated in this article, or claim that may be made by its manufacturer, is not guaranteed or endorsed by the publisher.

## Supplementary Material

Supplementary Table 1 Clinical score adapted from Ivester et al. (20)

Supplementary Figure 1 Cell counts of different populations per ml BALF calculated from cytospins or flow cytometry results. Counts of the main cell populations per ml BALF were calculated from the total nucleated cell counts and results of **(A–E)** cytospin microscopy analysis, or **(F–J)** flow cytometry analysis. Cell counts were similar between groups for **(A, F)** lymphocytes, **(C)** macrophages (Mph), **(D)** eosinophils, **(E)** mast cells, **(H)** CD14^+^ cells, **(I)** CD16^+^ cells, and **J)** IgE^+^CD14^-^ cells. Horses with sEA had significantly increased counts of PMN per ml BALF compared to healthy horses when calculated based on results from **B)** microscopic cytospin analysis or **(G)** from flow cytometry analysis (DH24A^+^ cells). All counts are presented in scatter plots with bars indicating group medians. Asterisks represent differences between groups with p<0.05 in the Kruskal-Wallis tests.

Supplementary Figure 2 Flow cytometry gating strategy for the main BAL cell populations. Gating strategy is shown for **(A)** BAL cells and **(B)** PBMC of one representative horse with severe equine asthma (sEA). Doublets and dead cells were excluded, and lymphocyte and non-lymphocyte populations were separated according to FSC-SSC characteristics. T cell sub-populations as well as Ig^+^CD4^-^CD8^-^ cells were gated from the lymphocyte population. Putative MC or basophils (IgE^+^CD14^-^), IgE-binding Mph and monocytes (IgE^+^CD14^+^), PMN (DH24A^+^), and macrophages (CD14^+^DH24A^-^) were gated from the non-lymphocyte population.

Supplementary Figure 3 Validation of flow cytometric polymorphonuclear (PMN) cell identification by MHCII staining. **(A)** Gating strategy is shown for BAL cells from one representative horse with severe equine asthma (sEA) and one healthy horse. PMN were gated from singlet live non-lymphocytes (NL) either by SSC-A vs. DH24A (DH24A^+^), or MHCII vs. DH24A^+^ (MHCII^lo^DH24A^+^) gating. The MHCII^-^DH24A^+^ population is also depicted as SSC-A vs. DH24A plot for comparison. **(B)** Percentages of DH24A^+^MHCII^-^ cells are plotted for BAL cells and PBMC. Results from individual horses are shown with bars indicating median values. Asterisks represent differences between groups with p<0.05 in Kruskal-Wallis tests. These results **(B)** match those from DH24A^+^ NL. mEA (mild to moderate equine asthma), sEA (severe equine asthma), tr (treated with steroids or bronchodilators), EA rem (equine asthma in remission).

Supplementary Figure 4 FACS sorted IgE^+^CD14^-^ reveal variable metachromatic staining in microscopic cytology analysis. Live BAL cells from three healthy horses were FACS sorted after hierarchical gating of **(A)** non-lymphocytes (NL) (FSC vs. SSC), **(B)** DH24A-negative NL, and then **(C)** IgE^+^CD14^-^ DH24A^-^ NL, after live staining as indicated in **(D)**. Composition of the cells is illustrated **(E)** before sorting and purity of **(F)** the sorted fraction after re-analysis by flow cytometry was 85%, 79%, and 78% IgE^+^CD14^-^ DH24A^-^ NL for samples from the three horses, respectively. **(G)** The IgE^+^CD14^-^ DH24A^-^ NL were analyzed for MHCII and were MHCII^lo^. Toluidine blue stained cytospins exemplified for one sample of **H**) the BAL cells pre-sort and **I**) the sorted cells confirmed enrichment of metachromatic cells (93%, 68%, and 77% by microscopic differentiation, respectively), but with variable degree of metachromatic granules per cell. Filled arrowheads indicate cells with many metachromatic granules, open arrowheads indicate cells with few metachromatic granules (sorted fraction I) in representative images.

Supplementary Figure 5 Flow cytometry gating strategy for macrophage subpopulations. Gating strategy is shown for **(A)** BAL cells and **(B)** PBMC of one representative healthy horse. Doublets, dead cells and lymphocytes were excluded as shown in **Supplementary Figure 1**. Next, DH24A^+^ PMN were excluded from the non-lymphocyte fraction. Macrophage subpopulations were analyzed by gating of CD14 against SSC-A, CD16 against SSC-A, or CD14 against CD16 to reveal double-positive cells.

Supplementary Figure 6 B cells are a small fraction of the CD4^-^CD8^-^Ig^+^ lymphocytes in BAL, but the majority in PBMC. **(A)** Percentages of CD4^-^CD8^-^ lymphocytes were similar between all groups in BAL cells, or PBMC (n=37 horses). **(B)** BAL cells and PBMC of seven horses (4 healthy, 2 mEA, 1 sEA) were additionally stained as indicated in the table to analyze if the CD4^-^CD8^-^Ig^+^ lymphocytes in BAL and PBMC are B cells and express CD79a (intracellular staining). Singlet live lymphocytes were gated in **(C)** BAL cells and **(F)** PBMC as indicated in a representative example (healthy). CD4^-^CD8^-^ lymphocytes were analyzed for Ig and CD79a expression, and the co-expression of these markers. Additionally, CD79a was plotted against SSC-A for the non-lymphocyte (NL) and lymphocyte **(L)** fractions. The percentages of different subsets in all lymphocytes were quantified in **(D)** BAL cells, and **(G)** PBMC (bars represent median). In PBMC, Ig and CD79a were co-expressed on most CD4^-^CD8^-^ lymphocytes and the percentages of Ig^+^ or CD79a^+^ lymphocytes corresponded in most samples **(H)**, while in BAL cells only few CD4^-^CD8^-^Ig^+^ lymphocytes also expressed CD79a and their percentages did not correspond **(E)**.

Supplementary Figure 7 CD154 and cytokines are incompletely co-expressed and T cells as well as CD4^-^CD8^-^ lymphocytes express cytokines in BAL and PBMC after *in vitro* stimulation with PMA and ionomycin. **(A–C)** *In vitro* stimulated BAL cells or PBMC (total n=26) singlet live lymphocytes were analyzed for co-expression of CD154 and T helper cytokines. Representative plots with quadrant gates on singlet live BAL lymphocytes from one horse with severe equine asthma (sEA) are shown and quantified net percentages per BAL or PBMC lymphocytes of **(A)** CD154^+^IFN-γ^+^, **(B)** CD154^+^IL-4^+^, and **(C)** CD154^+^IL-17A^+^ lymphocytes are depicted in bar graphs. Values from individual horses are shown as symbols with bars indicating median values per group (n=4–7). Differences between the groups were not statistically significant (p>0.12). **(D–H)** BAL cells (top) and PBMC (bottom) of three healthy horses were stimulated *in vitro* with PMA and ionomycin for 4 h, in comparison to medium incubation, fixed and stained for CD4 and CD8 surface markers and single (intracellular) cytokines with optimized antibody fluorophore combinations to differentiate cytokine-expressing T cells. Singlet live lymphocytes were gated and representative examples of PMA/ionomycin stimulated lymphocytes from one horses’ BAL and PBMC are shown. All cytokine-expressing lymphocytes were gated (small plots vs. SSC) and analyzed for CD4 vs. CD8 expression. All cytokines were expressed by T cells (CD4^+^ or CD8^+^) and by CD4^-^CD8^-^ lymphocytes in variable frequencies.

## Abbreviations

AadO_2_, alveolar to arterial oxygen pressure difference; BAL, bronchoalveolar lavage; BALF, bronchoalveolar lavage fluid; BD, bronchodilator; CD, cluster of differentiation; CTL, cytotoxic T lymphocytes; EA, equine asthma; FC, flow cytometry; HOARSI, horse owner assessed respiratory signs index; IL, interleukin; IFN, interferon; MC, mast cells; mEA, mild to moderate equine asthma; PBMC, peripheral blood mononuclear cells; PBS, phosphate buffered saline; PMA, Phorbol-12-myristat-13-acetat; PMN, polymorphonuclear cells; EA rem, equine asthma in remission; sEA, severe equine asthma; ST, steroids; Th, T helper; TNF, tumor necrosis factor.
