# Supplementary material for: Comprehensive Flow Cytometric Characterization of Bronchoalveolar Lavage Cells Indicates Comparable Phenotypes Between Asthmatic and Healthy Horses But Functional Lymphocyte Differences
Source: Front Immunol. 2022 Jul 6;13:896255. doi: 10.3389/fimmu.2022.896255 (PMC9296846; doi:10.3389/fimmu.2022.896255)
Supplement: Supplementary Table 1 — Clinical score adapted from Ivester et al. (20) [file Table_1.docx]

**Supplementary table 1 Clinical score adapted from Ivester et al.** (20)

| **Parameter** | **Range** | **Description** |
| --- | --- | --- |
| Nasal discharge (ND) | 0-3 | 0 no ND or serous  1 mild mucous ND  2 moderate mucopurulent ND  3 severe mucopurulent ND |
| Nasal flare (NF) | 0-3 | 0 physiological nostril movement  1 inspiratory NF  2 inspiratory and expiratory NF, visible movement of the nostrils  3 inspiratory and expiratory NF, no movement of the nostrils |
| Cough | 0-3 | 0 none or 1x provoked after trachea compression  1 several provoked cough  2 spontaneous cough  3 coughing attack lasting > 30 seconds |
| Respiratory Rate | 0-3 | 0 <16/minute  1 16-20/minute  2 21-30/minute  3 >30/minute |
| Abdominal lift | 0-3 | 0 hardly abdominal effort  1 slight expiratory flattening of the ventral flank  2 obvious abdominal flattening, heave line maximum extending halfway between cubital joint and *tuber coxae*  3 obvious abdominal lift, heave line extending beyond halfway between cubital joint and *tuber coxae* |
| Lung auscultation at rest | 0-2 | 0 physiological lung sounds  1 increased sounds louder than physiological trachea  2 crackles, wheezes |
| Lung auscultation after re-breathing | 0-2 | 0 physiological lung sounds  1 increased sounds louder than physiological trachea  2 crackles, wheezes |
| **Score sum** | **0-19** | **Sum of all scores above** |
