# Supplementary figures and images for: Comprehensive Flow Cytometric Characterization of Bronchoalveolar Lavage Cells Indicates Comparable Phenotypes Between Asthmatic and Healthy Horses But Functional Lymphocyte Differences
Source: Front Immunol. 2022 Jul 6;13:896255. doi: 10.3389/fimmu.2022.896255 (PMC9296846; doi:10.3389/fimmu.2022.896255)

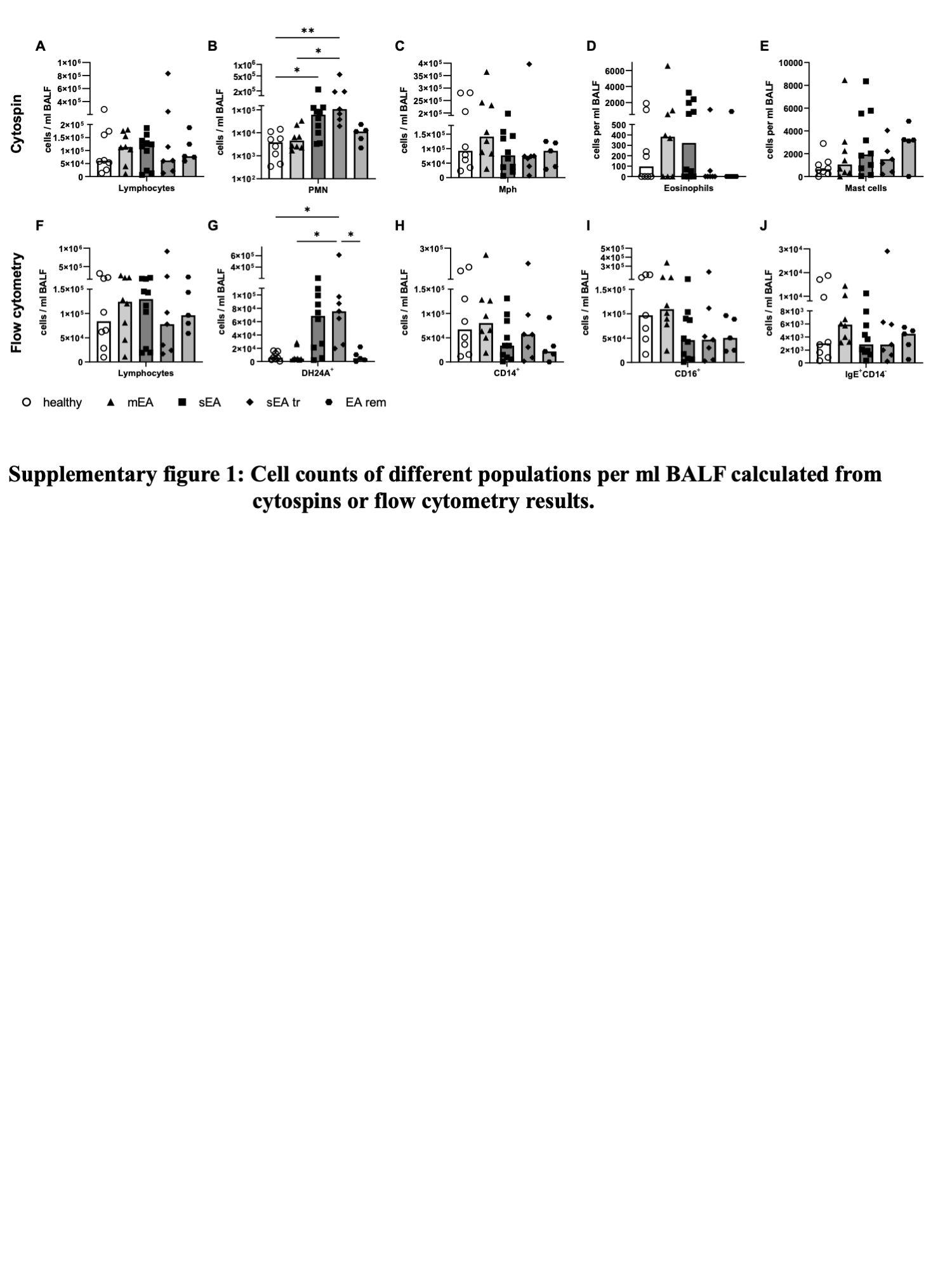

Supplement: Supplementary Figure 1 — Cell counts of different populations per ml BALF calculated from cytospins or flow cytometry results. Counts of the main cell populations per ml BALF were calculated from the total nucleated cell counts and results of (A–E) cytospin microscopy analysis, or (F–J) flow cytometry analysis. Cell counts were similar between groups for (A, F) lymphocytes, (C) macrophages (Mph), (D) eosinophils, (E) mast cells, (H) CD14+ cells, (I) CD16+ cells, and J) IgE+CD14- cells. Horses with sEA had significantly increased counts of PMN per ml BALF compared to healthy horses when calculated based on results from B) microscopic cytospin analysis or (G) from flow cytometry analysis (DH24A+ cells). All counts are presented in scatter plots with bars indicating group medians. Asterisks represent differences between groups with p<0.05 in the Kruskal-Wallis tests. [file Image_1.tiff]

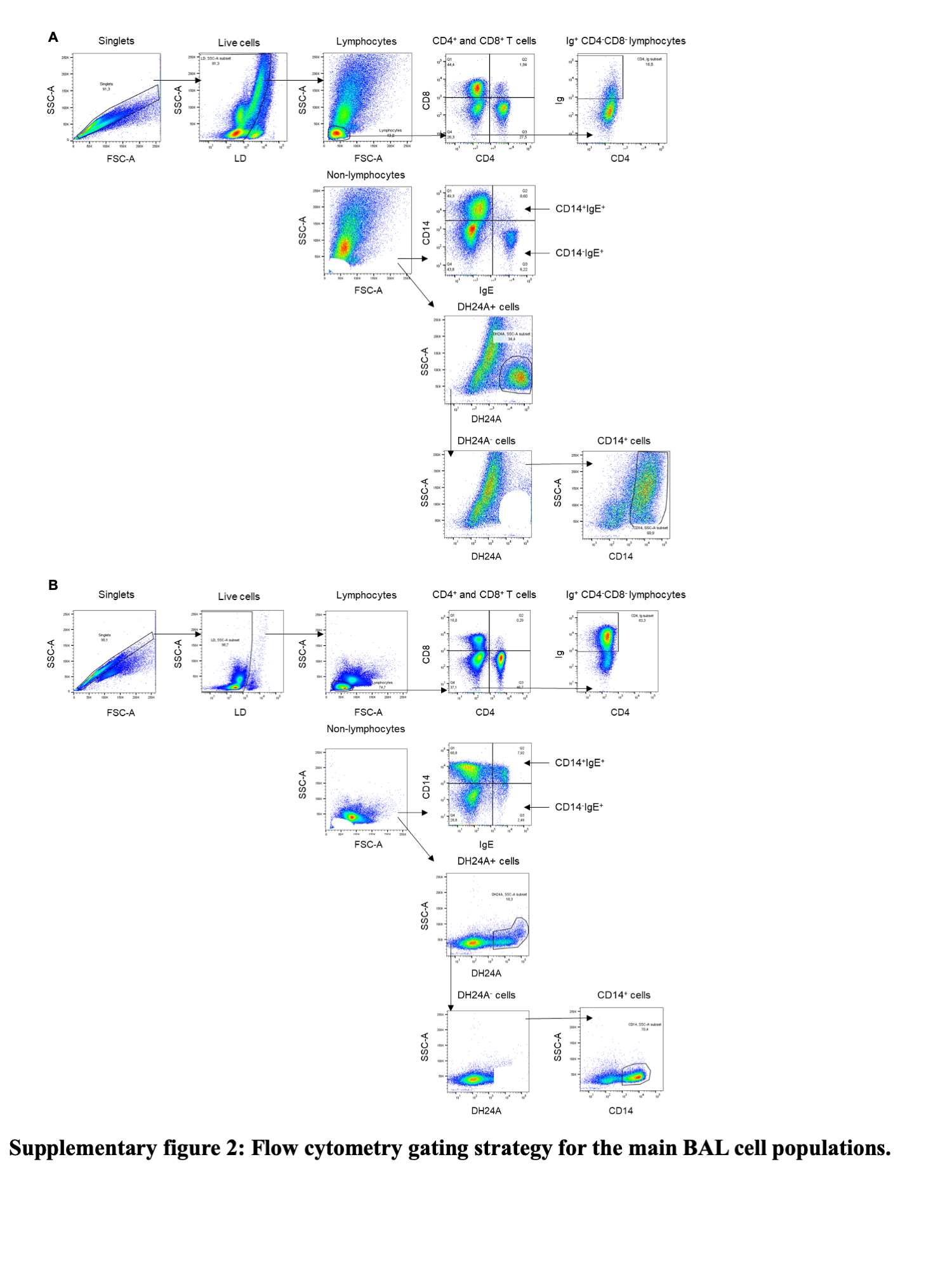

Supplement: Supplementary Figure 2 — Flow cytometry gating strategy for the main BAL cell populations. Gating strategy is shown for (A) BAL cells and (B) PBMC of one representative horse with severe equine asthma (sEA). Doublets and dead cells were excluded, and lymphocyte and non-lymphocyte populations were separated according to FSC-SSC characteristics. T cell sub-populations as well as Ig+CD4-CD8- cells were gated from the lymphocyte population. Putative MC or basophils (IgE+CD14-), IgE-binding Mph and monocytes (IgE+CD14+), PMN (DH24A+), and macrophages (CD14+DH24A-) were gated from the non-lymphocyte population. [file Image_2.tiff]

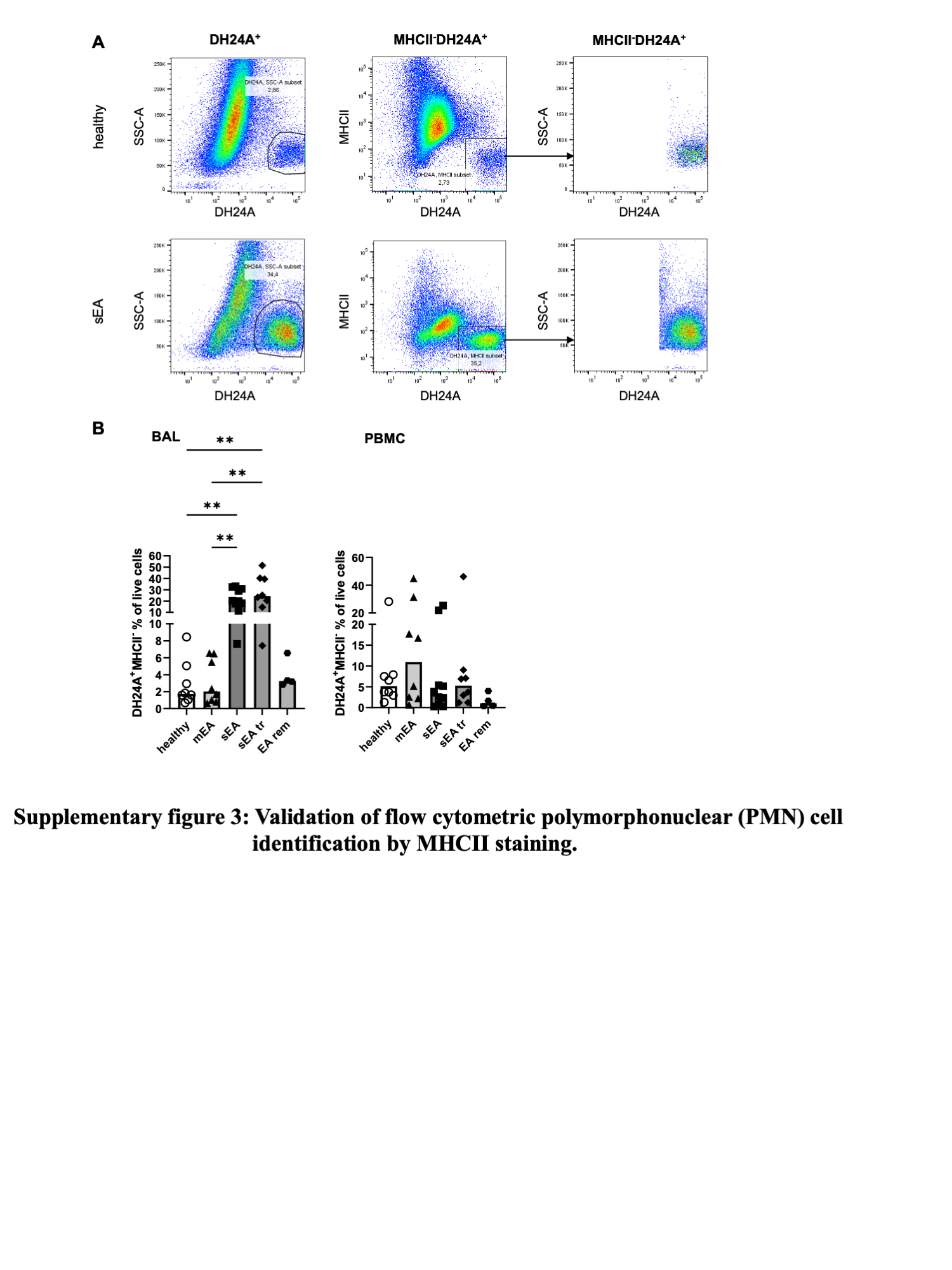

Supplement: Supplementary Figure 3 — Validation of flow cytometric polymorphonuclear (PMN) cell identification by MHCII staining. (A) Gating strategy is shown for BAL cells from one representative horse with severe equine asthma (sEA) and one healthy horse. PMN were gated from singlet live non-lymphocytes (NL) either by SSC-A vs. DH24A (DH24A+), or MHCII vs. DH24A+ (MHCIIloDH24A+) gating. The MHCII-DH24A+ population is also depicted as SSC-A vs. DH24A plot for comparison. (B) Percentages of DH24A+MHCII- cells are plotted for BAL cells and PBMC. Results from individual horses are shown with bars indicating median values. Asterisks represent differences between groups with p<0.05 in Kruskal-Wallis tests. These results (B) match those from DH24A+ NL. mEA (mild to moderate equine asthma), sEA (severe equine asthma), tr (treated with steroids or bronchodilators), EA rem (equine asthma in remission). [file Image_3.tiff]

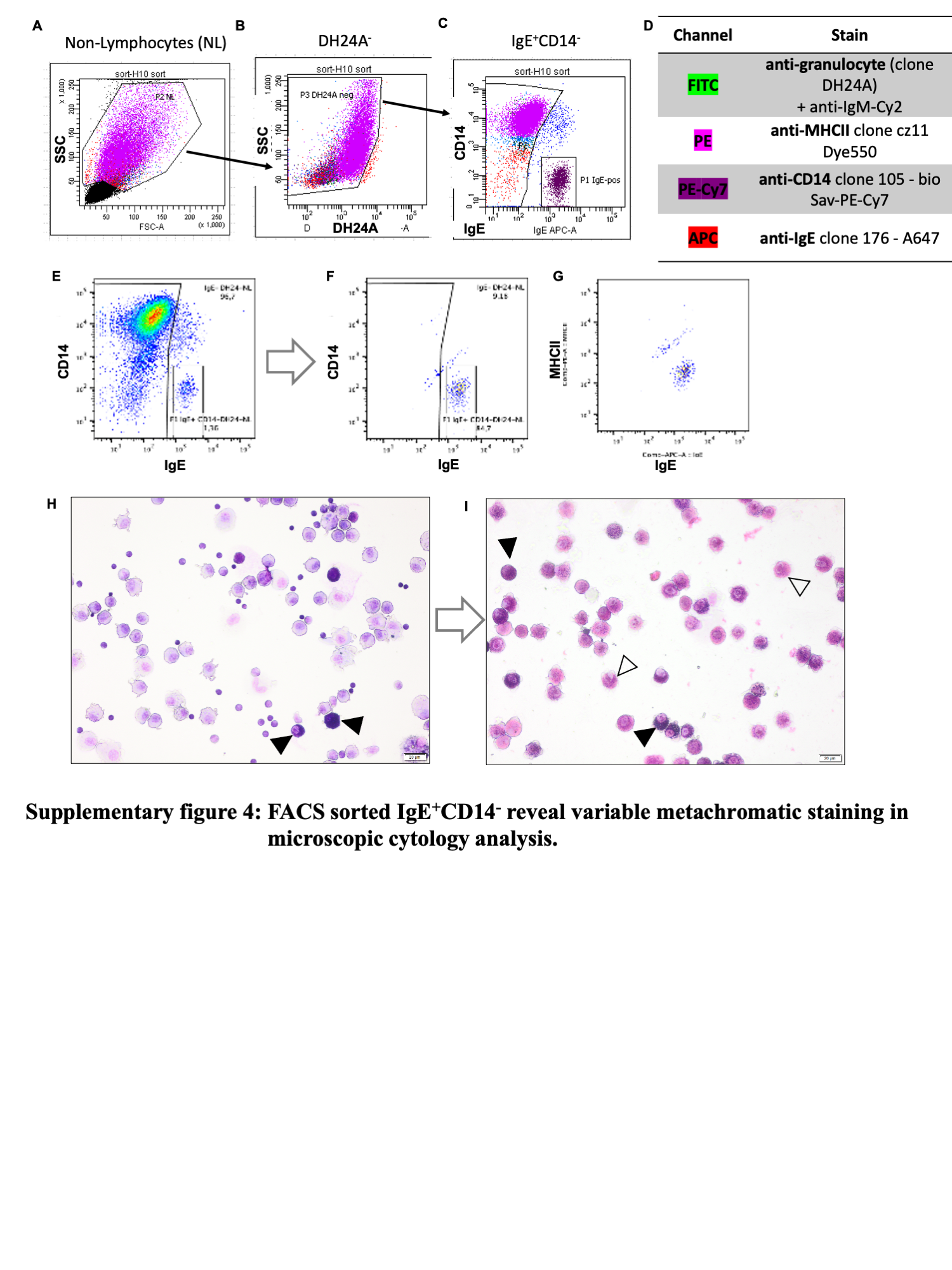

Supplement: Supplementary Figure 4 — FACS sorted IgE+CD14- reveal variable metachromatic staining in microscopic cytology analysis. Live BAL cells from three healthy horses were FACS sorted after hierarchical gating of (A) non-lymphocytes (NL) (FSC vs. SSC), (B) DH24A-negative NL, and then (C) IgE+CD14- DH24A- NL, after live staining as indicated in (D). Composition of the cells is illustrated (E) before sorting and purity of (F) the sorted fraction after re-analysis by flow cytometry was 85%, 79%, and 78% IgE+CD14- DH24A- NL for samples from the three horses, respectively. (G) The IgE+CD14- DH24A- NL were analyzed for MHCII and were MHCIIlo. Toluidine blue stained cytospins exemplified for one sample of H) the BAL cells pre-sort and I) the sorted cells confirmed enrichment of metachromatic cells (93%, 68%, and 77% by microscopic differentiation, respectively), but with variable degree of metachromatic granules per cell. Filled arrowheads indicate cells with many metachromatic granules, open arrowheads indicate cells with few metachromatic granules (sorted fraction I) in representative images. [file Image_4.tiff]

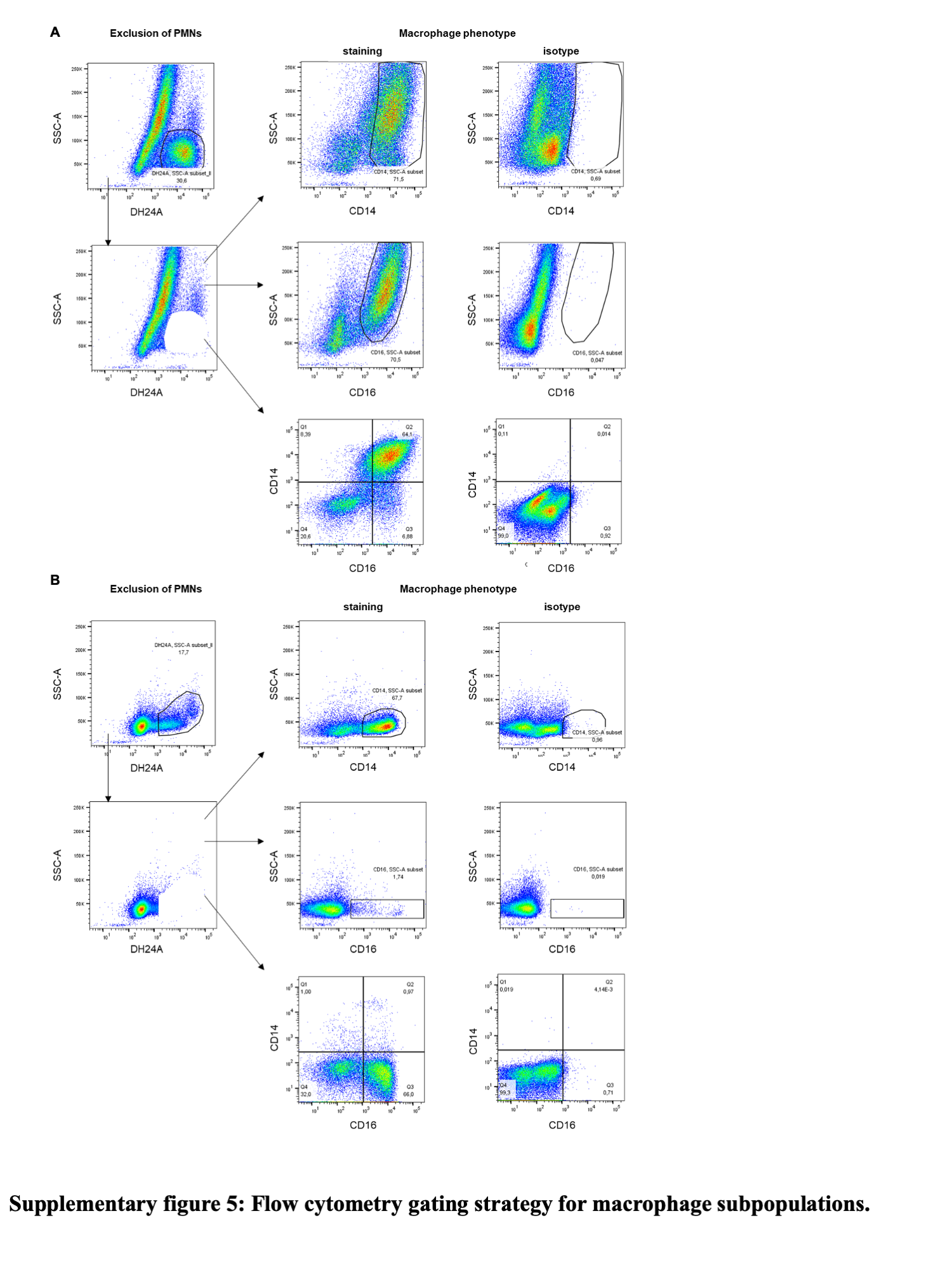

Supplement: Supplementary Figure 5 — Flow cytometry gating strategy for macrophage subpopulations. Gating strategy is shown for (A) BAL cells and (B) PBMC of one representative healthy horse. Doublets, dead cells and lymphocytes were excluded as shown in Supplementary Figure 1 . Next, DH24A+ PMN were excluded from the non-lymphocyte fraction. Macrophage subpopulations were analyzed by gating of CD14 against SSC-A, CD16 against SSC-A, or CD14 against CD16 to reveal double-positive cells. [file Image_5.tiff]

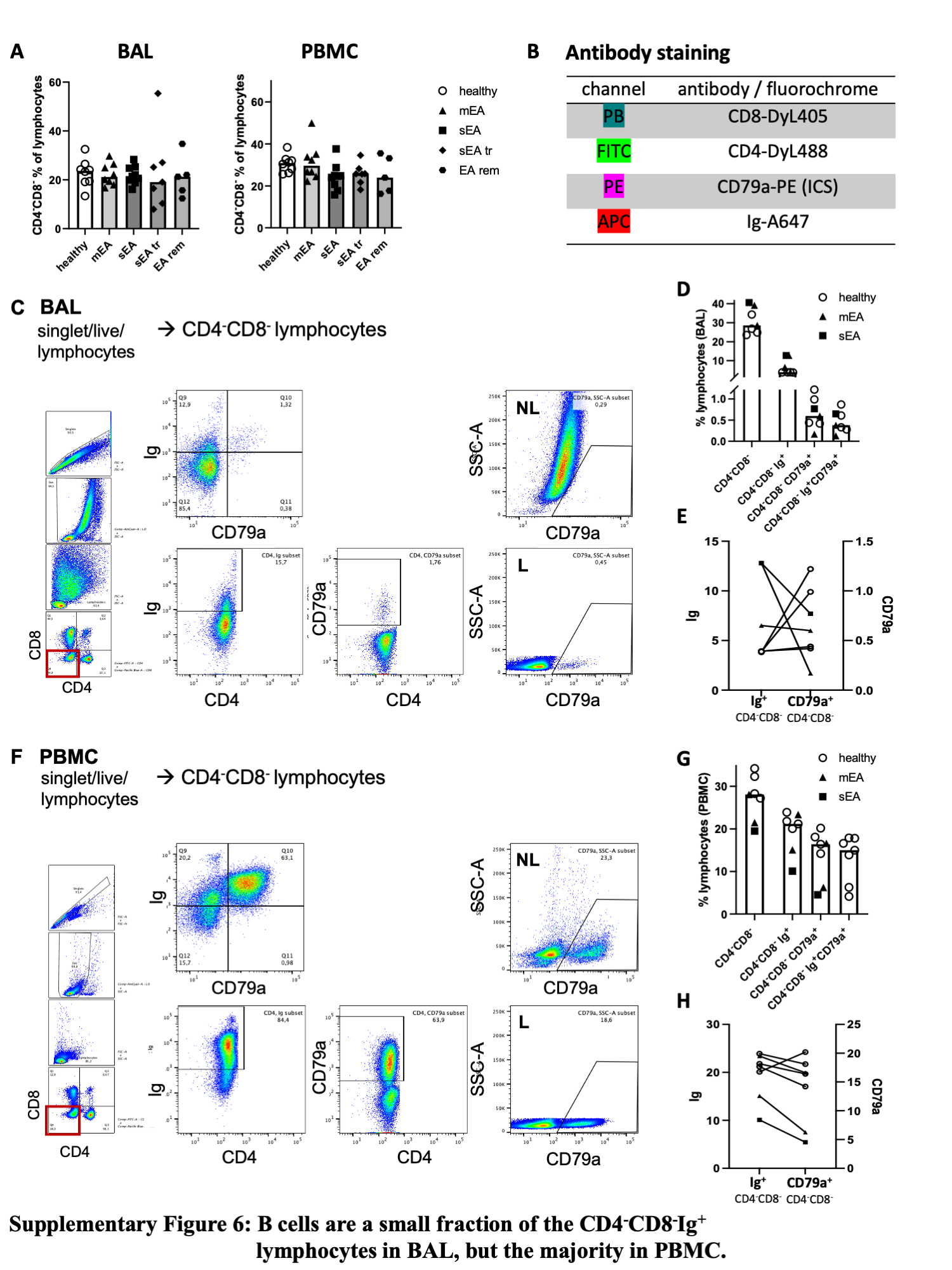

Supplement: Supplementary Figure 6 — B cells are a small fraction of the CD4-CD8-Ig+ lymphocytes in BAL, but the majority in PBMC. (A) Percentages of CD4-CD8- lymphocytes were similar between all groups in BAL cells, or PBMC (n=37 horses). (B) BAL cells and PBMC of seven horses (4 healthy, 2 mEA, 1 sEA) were additionally stained as indicated in the table to analyze if the CD4-CD8-Ig+ lymphocytes in BAL and PBMC are B cells and express CD79a (intracellular staining). Singlet live lymphocytes were gated in (C) BAL cells and (F) PBMC as indicated in a representative example (healthy). CD4-CD8- lymphocytes were analyzed for Ig and CD79a expression, and the co-expression of these markers. Additionally, CD79a was plotted against SSC-A for the non-lymphocyte (NL) and lymphocyte (L) fractions. The percentages of different subsets in all lymphocytes were quantified in (D) BAL cells, and (G) PBMC (bars represent median). In PBMC, Ig and CD79a were co-expressed on most CD4-CD8- lymphocytes and the percentages of Ig+ or CD79a+ lymphocytes corresponded in most samples (H), while in BAL cells only few CD4-CD8-Ig+ lymphocytes also expressed CD79a and their percentages did not correspond (E). [file Image_6.tiff]

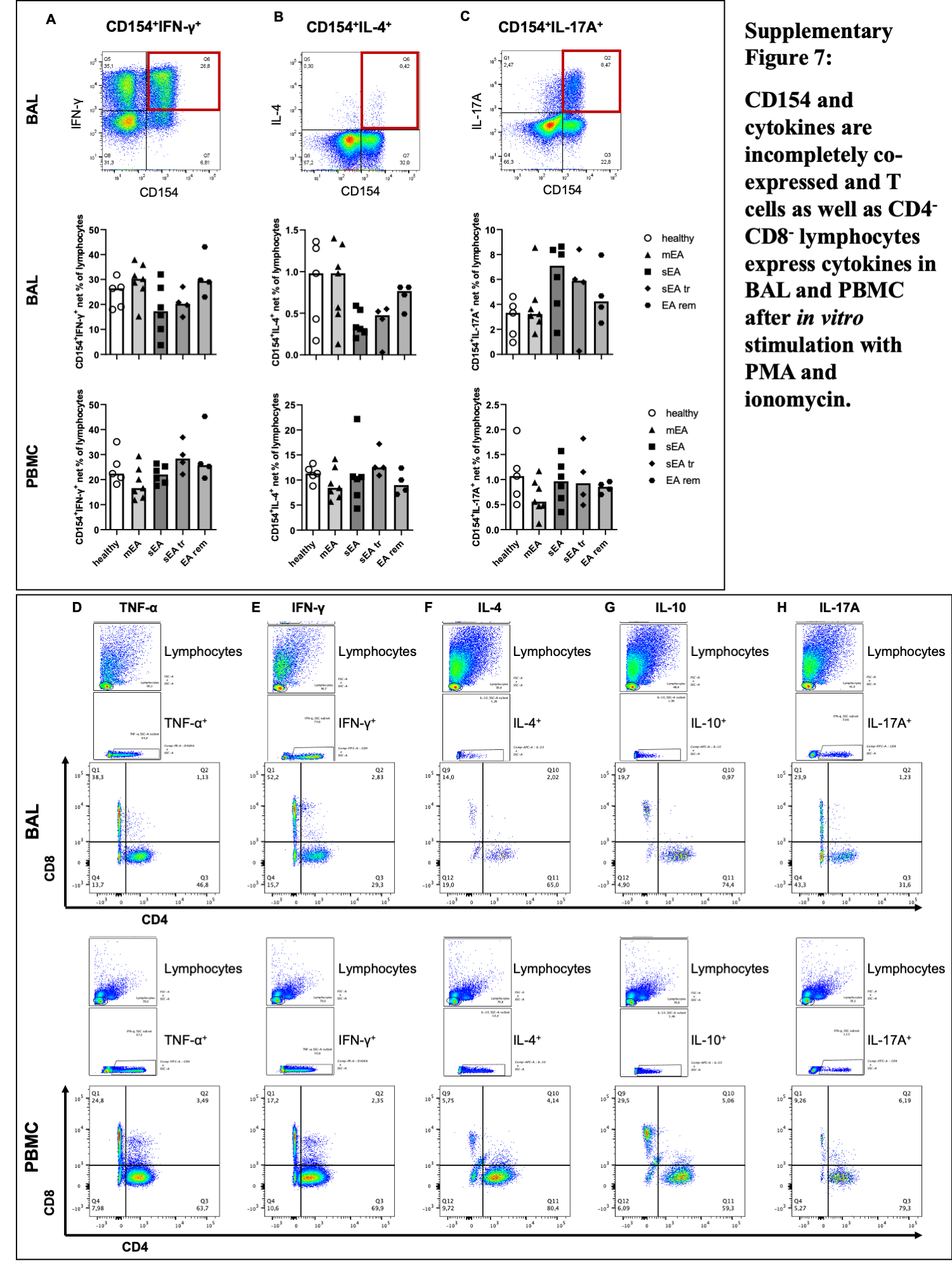

Supplement: Supplementary Figure 7 — CD154 and cytokines are incompletely co-expressed and T cells as well as CD4-CD8- lymphocytes express cytokines in BAL and PBMC after in vitro stimulation with PMA and ionomycin. (A–C) In vitro stimulated BAL cells or PBMC (total n=26) singlet live lymphocytes were analyzed for co-expression of CD154 and T helper cytokines. Representative plots with quadrant gates on singlet live BAL lymphocytes from one horse with severe equine asthma (sEA) are shown and quantified net percentages per BAL or PBMC lymphocytes of (A) CD154+IFN-γ+, (B) CD154+IL-4+, and (C) CD154+IL-17A+ lymphocytes are depicted in bar graphs. Values from individual horses are shown as symbols with bars indicating median values per group (n=4–7). Differences between the groups were not statistically significant (p>0.12). (D–H) BAL cells (top) and PBMC (bottom) of three healthy horses were stimulated in vitro with PMA and ionomycin for 4 h, in comparison to medium incubation, fixed and stained for CD4 and CD8 surface markers and single (intracellular) cytokines with optimized antibody fluorophore combinations to differentiate cytokine-expressing T cells. Singlet live lymphocytes were gated and representative examples of PMA/ionomycin stimulated lymphocytes from one horses’ BAL and PBMC are shown. All cytokine-expressing lymphocytes were gated (small plots vs. SSC) and analyzed for CD4 vs. CD8 expression. All cytokines were expressed by T cells (CD4+ or CD8+) and by CD4-CD8- lymphocytes in variable frequencies. [file Image_7.tiff]
